# Ostracism of an Albino Individual by a Group of Pigmented Catfish

**DOI:** 10.1371/journal.pone.0128279

**Published:** 2015-05-27

**Authors:** Ondřej Slavík, Pavel Horký, Matúš Maciak

**Affiliations:** 1 Department of Zoology and Fisheries, Faculty of Agrobiology, Food and Natural Resources, Czech University of Life Sciences Prague, Kamýcká 129, Prague, Czech Republic; 2 Department of Mathematical and Statistical Sciences, University of Alberta, Edmonton, Alberta, Canada; University of Leicester, UNITED KINGDOM

## Abstract

Physiological and behavioural constraints hinder albino individuals. Albino animals are rare in the wild; this trait is associated with easy detection by predators, non-native or damaged environments, and exclusively aphotic environments in total darkness. The social aspect of albinism is reported only for human beings, and the effect is distinguishable in time and space when social benefits, are used to a limited the extent. Thus far, the social consequences of albinism for animals remain unknown. We used socially established groups of the pigmented catfish, (*Silurus glanis*), to observe space and temporal distance detachment of albino specimens in laboratory conditions. The albino fish were separated at larger distances from the group than pigmented individuals with the same social status determined by familiarity, and this asymmetry also varied in time. Albinism-related ostracism results in a solitary existence, usually followed by enhanced predation risk. The motivation for an individual’s exclusion from a group appears to be the avoidance of the predation risk that increases not only for an odd individual but also for conspecifics within a group. Our findings indicate a role for albinism in behavioural processes related to sociality in a group of conspecifics.

## Introduction

Physiological and behavioural constraints hinder albino individuals [1,2]. Albino animals are rare in the wild; this trait is associated with easy detection by predators [3], non-native or damaged environments [3,4], and exclusively aphotic environments in total darkness [5]. Albinism encompasses genetically determined diseases that involve disorders of the melanin system, resulting in hypopigmentation [2]. Oculocutaneous albinism (OCA) is the result of a recessive mutation of the gene encoding the tyrosinase enzyme and is characterised by different levels of skin, hair, and eye pigmentation. Albinism has been recorded in plants [6], invertebrates [7], vertebrates, and human beings. The pleiotropic effect of albinism is considered to be responsible for physiological and behavioural disadvantages [2,6,8,9]. However, the social consequences of albinism have only been described for human beings during ontogenesis [10], which are displayed via religion stigmatisation and community ostracism [11]. Apparently, only limited participation in social benefits such as friendships, marriages, and carrier opportunities [11,12] has been recorded among albino people, suggesting that ostracism is distinguishable in time and space. Here, we show albino-biased ostracism in European catfish, (*Silurus glanis* L.), as the first evidence of the social consequences of albinism in animals. Familiar fish with former social experience show effective resource exploitation and protection from predators [13,14,15]. Catfish live in groups [16], and a group of familiar individuals is able to make a collective decision [13]. We used a group of familiar pigmented catfish to test their interaction with unfamiliar albino and pigmented conspecifics.

## Material and Methods

### Experimental animals

The fish used in the experiment were hatchery-reared juvenile catfish. Two shoals of pigmented and one shoal of albino catfish unfamiliar with each other were obtained from local fish suppliers (Czech Fishery Ltd., Rybářství Třeboň and Rybářství Nové Hrady, Czech Republic) to ensure that the individuals belonging to the distinct shoals had never been in contact. A total of 600 equally sized fish (200 from each shoal) were transported from the hatchery to the laboratory at the age of 4 months. The fish were transported under stable conditions in oxygenated tanks in an air-conditioned loading space, and the transport lasted approximately 2 hours. There was no observable effect of the transport on the health or mortality of the fish.

The fish were subsequently kept in 3 separate holding tanks (1000 L each, initial density 1.8 kg m^−3^; one shoal, i.e., 200 individuals per tank) for 6 weeks prior to the start of the experiment. The fish were fed food pellets ad libitum (Biomar Group, Denmark, www.biomar.com) distributed across the entire tank, providing free access to food to all individuals twice a day. The fish were kept under a natural photoperiod, maintaining the same regime to which they had become accustomed in the hatchery. The water was purified using biological filters with an integrated UV sterilizer (Pressure-Flo 5000, Rolf C. Hagen Inc., www.lagunaponds.com). The water temperature, dissolved oxygen and pH were controlled automatically (HOBO data logger; Onset Computer Corporation, Bourne, Massachusetts, USA).

The fish were tagged 10 days prior to the start of the experiment. The fish were anaesthetized with 2-phenoxyethanol (0.2 ml L^−1^; Merck KGaA, Germany) and then measured (standard length LS; mean 99 mm, range 81–117 mm) and weighed (mean body mass 9.1 g, range 4–16 g); no size differences between the shoals of fish were detected (standard length P > 0.82, n = 600; body mass P > 0.68, n = 600). Passive integrated transponders (PIT; Trovan ID 100, 0.1 g in air, 12 mm 2.1 mm; EID Aalten B.V., Aalten, Netherlands) were inserted into the abdominal cavity using a syringe. This method has been successfully used in behavioural experiments [17]. No adverse effects of PIT implantation or anaesthesia were observed.

All experimental fish (600 individuals) survived; after the experiment, the fish were released under the control of Fish Management Authorities into fish ponds with extensive production management.

### Experimental design

**Fig 1:**
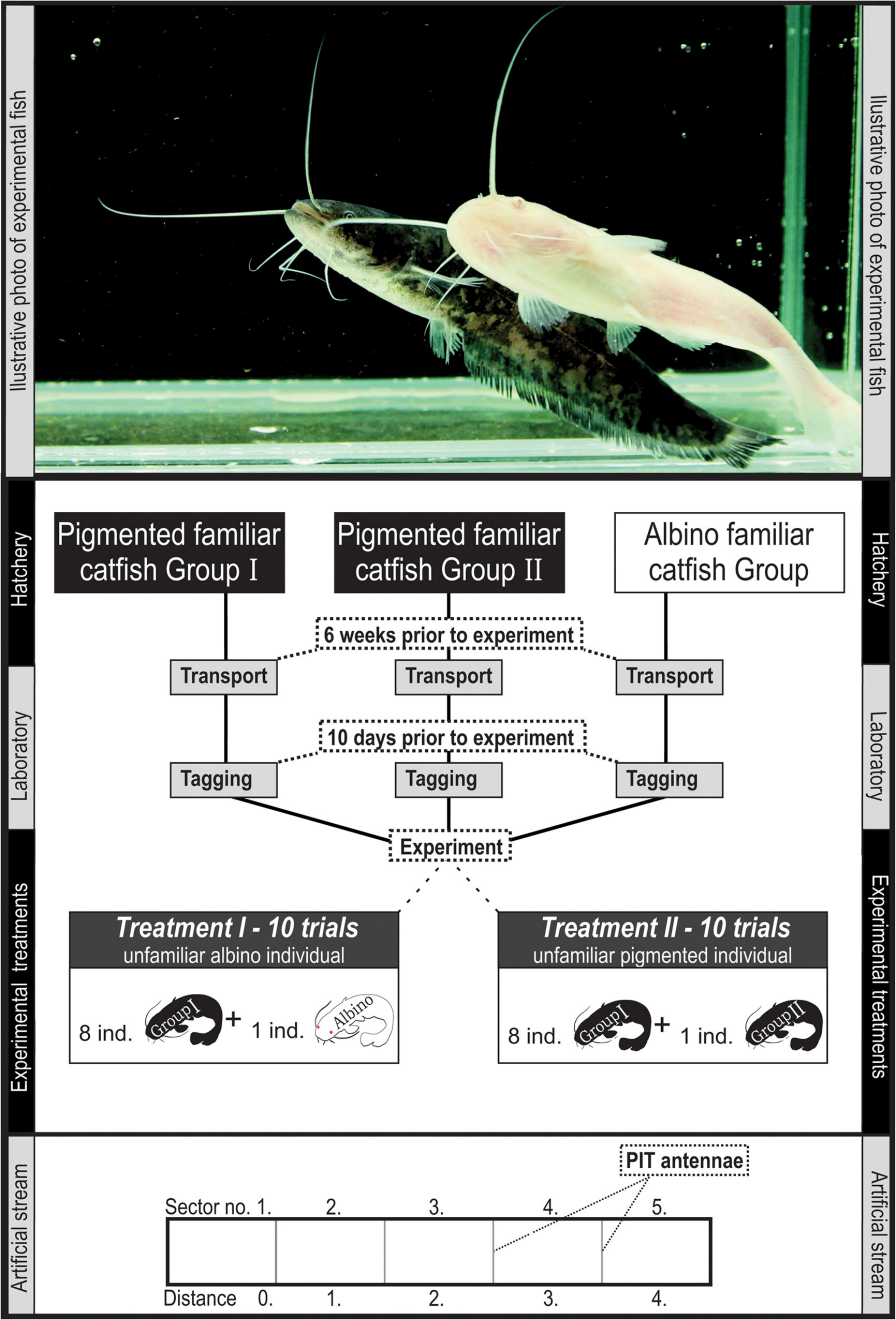
Illustrative figure of the experimental design and the artificial stream.

The laboratory experiment was conducted between November 15 and December 17, 2010, in an oval artificial stream (see [18] for details). For the purpose of this experiment, only the straight part of the stream was used (beige colour, 3.75 m long, 0.49 m wide and 0.32 m deep). This segment was divided into 5 subunits using 7 equidistant PIT antennae (Fig 1), and mesh was placed over the outer antennae to prevent the fish from escaping from the observed stream segment. The antennae (inner area 0.49 m × 0.25 m) were designed to serve as frames for the detection of fish swimming through them and were connected to a recorder that stored the detection information (PIT tag code, date, time and antenna number) in its internal memory. The handling conditions were comparable to those in the holding tanks, and the water quality and flow were controlled by 2 Pressure-Flo 5000 units (60 L/min each). This arrangement generated a visible current (0.01 m s^−1^) circulating through the stream; however, the fish did not have to swim continuously to maintain their positions.

A group of 8 familiar pigmented catfish was tested for its ability to co-opt an albino (first treatment) or pigmented (second treatment) unfamiliar conspecific in an artificial stream (Fig 1). The treatments were rotated regularly (one day an albino; the other day a pigmented treatment), and every treatment was repeated ten times, resulting in twenty individual trial repetitions. Every experimental trial lasted for 24 h (beginning at 8:00 a.m.). As individual fish were not used repeatedly in the experiment, 180 catfish were used during the entire experiment.

### Data analyses

Data analyses were performed using R software (R Development Core Team, 2010, version 2.11.1). The ‘distance’ between the additional unfamiliar catfish and each of eight individuals from the familiar group was computed as the number of artificial channel sectors between them. A distance equalling zero means that both the evaluated individuals are in the same sector; while a distance equalling four means that the evaluated individuals are separated in the most distant sectors (Fig 1). The ‘average distance’ is the mean of all ‘distance’ values between unfamiliar catfish and members of the familiar group and can be real values from 0 (all fish in one artificial stream sector together) up to 4 (all members from the familiar group in one sector at one end of the channel, with the additional unfamiliar individual at the opposite end of the channel). ‘Isolation’ was defined as a binary variable; 1 means that the additional unfamiliar catfish is isolated in one artificial stream sector; 0 means that there is at least one conspecific from a familiar group present. Another binary variable ‘cohesion’ was used to describe whether the group of familiar pigmented catfish is cohesive (i.e., distributed in three or less artificial stream sectors; value 1) or not (i.e., distributed in more than three artificial stream sectors; value 0).

To avoid the dependence of two consecutive points in raw continuous data (a type of M-dependence structure), a regular 10-minute grid approach was applied, resulting in 2880 lines for the final dataset.

### Statistical analyses

Statistical analyses were performed using the SAS software package (version 9.2). Linear mixed effect modelling was applied to analyse the average distance between the unfamiliar catfish and members of the familiar group. The MIXED procedure with a random intercept term to account for variability among the trials was used for this purpose [19]. The final model was built on the basis of the stepwise forward procedure (all two way interactions were tested), always according to a better Akaike’s Information Criterion (AIC) value and considering that the model with the lower AIC fit the data better [20]. The parameter estimates together with the standard error estimates and the appropriate significance tests of the final model are given in Table 1.

**Table 1:** Parameter estimates with corresponding standard errors and p-values for the proposed mixed model.

Parameter	Factor level	Estimate	St. Error	P-value
Intercept	-	0.9873	0.0839	< 0.0001
Time (minutes)	-	0.0011	< 0.0003	0.0041
Time squared (minutes)	-	-2.97 x10^-7	< 0.0001	< 0.0001
Time cubed (minutes)	-	-278 x10^-12	< 0.0001	< 0.0001
Treatment	Pigmented catfish	-0.3851	0.0893	< 0.0001
	Albino catfish	0	-	-
Time x Treatment	Pigmented catfish	214 x10^-6	0.0001	0.0036
	Albino catfish	0	-	-

Time covariate enters the model as a third order polynom (Time, Time squared and Time cubed).

Additional binomial distributional data were subjected to a χ^2^ test to evaluate i) the effect of albinism on the ‘isolation’ of unfamiliar catfish in an artificial stream sector and ii) the ‘cohesion’ (i.e., distribution in three or less artificial stream sectors) of the group of familiar pigmented catfish in the presence of an albino or pigmented unfamiliar conspecific. The FREQ procedure was used for this purpose, designing it to compare the frequency of ‘isolation’ and ‘cohesion’ occurrence under different treatments. Relative deviations from the hypothesized values (equal proportions, i.e. 50% occurrence in this case) were used to express the character of the relationships. The relative deviation for a level is the difference between the observed and hypothesized/expected test percentage occurrence divided by the test percentage occurrence.

The GENMOD procedure with binomial distributions was designed to estimate the probability of ‘cohesion’ occurrence. The explanatory variable tested was the average distance between unfamiliar catfish and members of the familiar group across different treatments (albino or pigmented unfamiliar individual). We applied an analysis of repeated measurements based on the generalised estimating equation (GEE) approach [21], which is an extension of a generalised linear model and provides a semi-parametric approach to longitudinal data analysis. To account for repeated measures during the same trials, we used a REPEATED statement with the trial defined in the SUBJECT option. Data entering the statistical analyses are in the supporting information file **S1 Table**.

### Ethics statement

All of the laboratory experimental procedures complied with valid legislative regulations (law no. 246/1992, §19, art. 1, letter c); the permit was subjected to O. Slavík, qualified according to Law no. 246/1992, §17, art. 1; permit no. CZ00167. All laboratory sampling including PIT implantation was carried out with the relevant permission from the Departmental Expert Committee for authorization experimental project of the Ministry of Education, Youth and Sports of the Czech Republic (permit no. MSMT-31220/2014-6, registered by the Ministry of Education, Youth and Sports of the Czech Republic). The study did not involve endangered or protected species.

## Results

**Fig 2:**
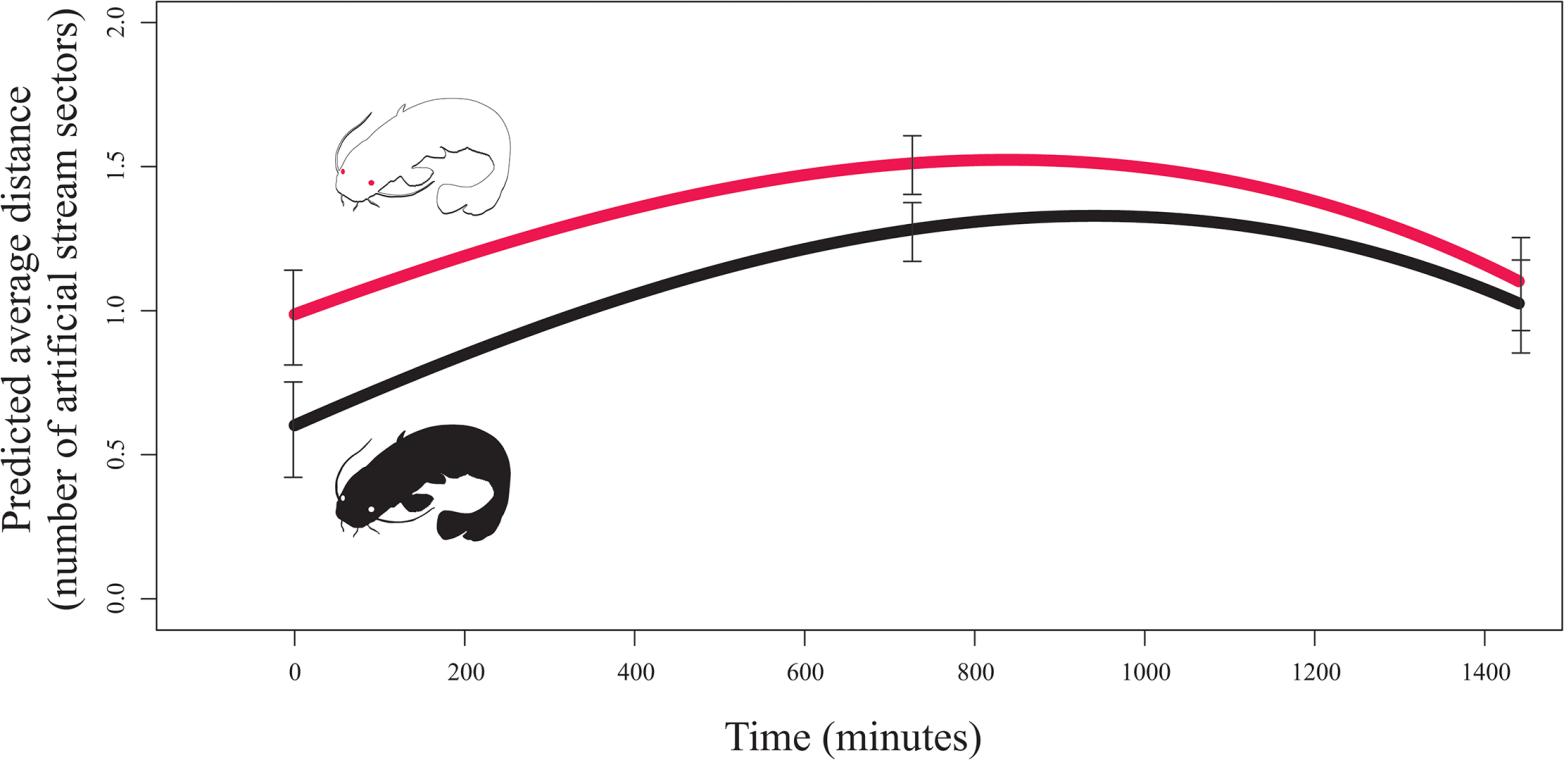
The distance between experimental individual and the fish group showed as predicted average distance between unfamiliar catfish and members of a familiar group across two treatments (n = 2880). Red line = an albino unfamiliar conspecific; Black line = a pigmented unfamiliar conspecific. Standard errors for the start, mid and end of the experiment is provided.

**Fig 3:**
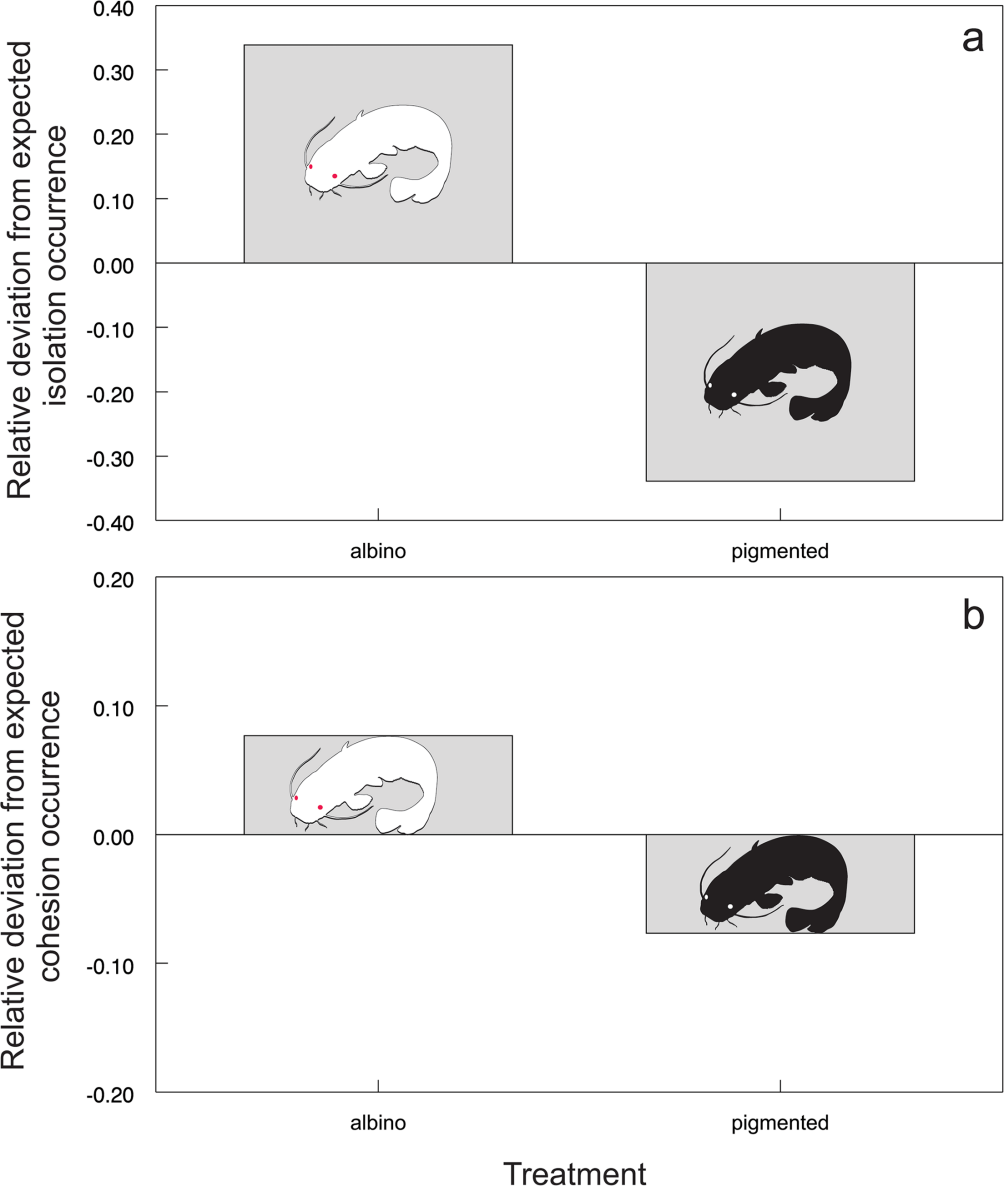
Probability of experimental fish isolation showed as relative deviation from the expected occurrence of isolation (a) and probability of group cohesion showed as relative deviation from the expected occurrence of cohesion (b) across different treatments. Zero represents the test equal occurrence (i.e., 50% occurrence in this case).

An albino individual was found to be more distanced from the group of pigmented fish, and this relationship evolved over time (Table 1; Fig 2). The probability that an albino fish would be isolated in a separate sector of the artificial stream was two times higher than that for a pigmented individual (χ^2^ = 70.1293, P<0.001; Fig 3A). Furthermore, the group of familiar catfish was more cohesive in the presence of an albino (χ^2^ = 7.5031, P<0.01; Fig 3B), suggesting cohesion as a possible strategy to avoid the albino fish.

**Fig 4:**
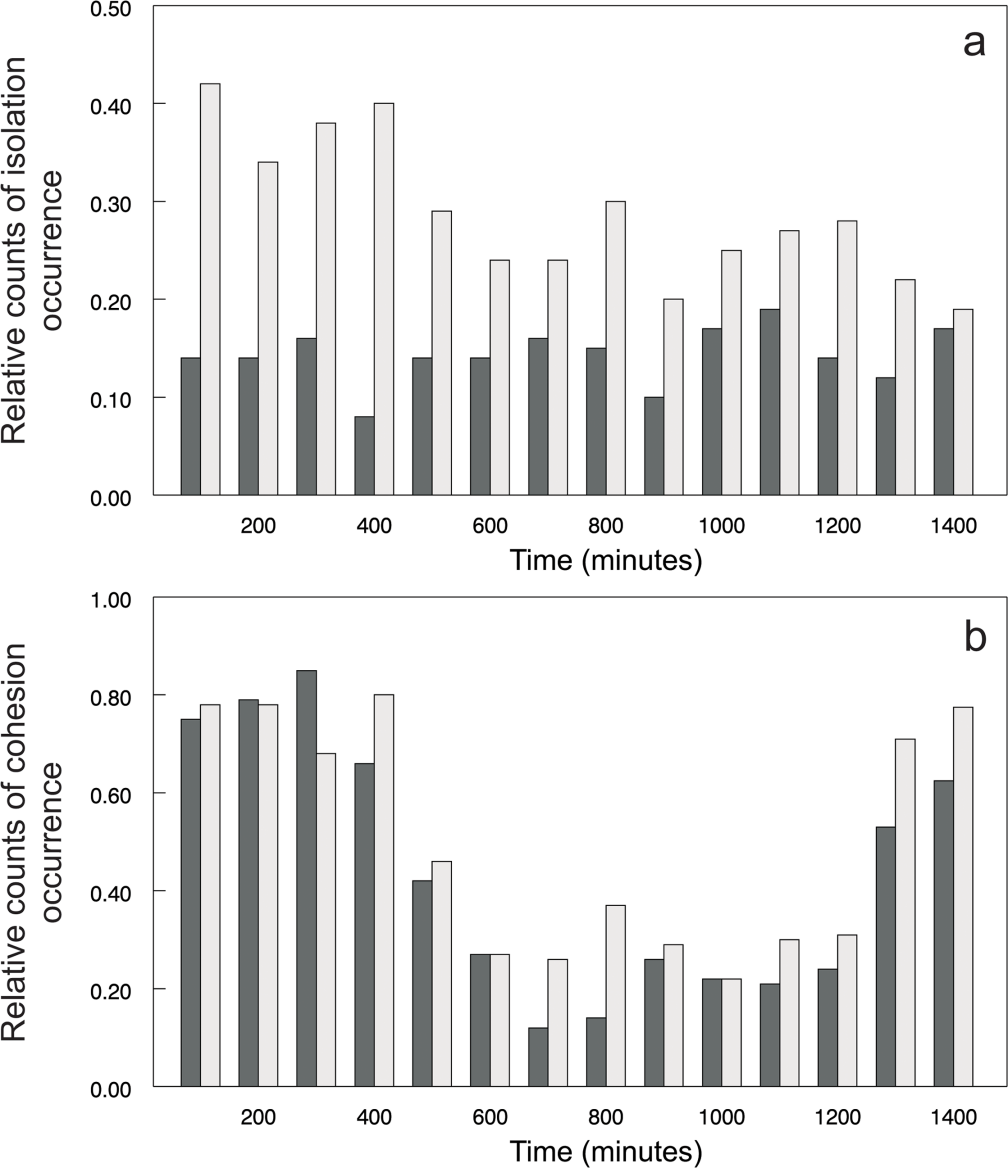
Changes in fish isolation and group cohesion across time showed as a relative count distributions during the given time domain with respect to a chance of isolation (a) and cohesion (b) occurrence. White bars = an albino unfamiliar conspecific; Black bars = a pigmented unfamiliar conspecific.

**Fig 5:**
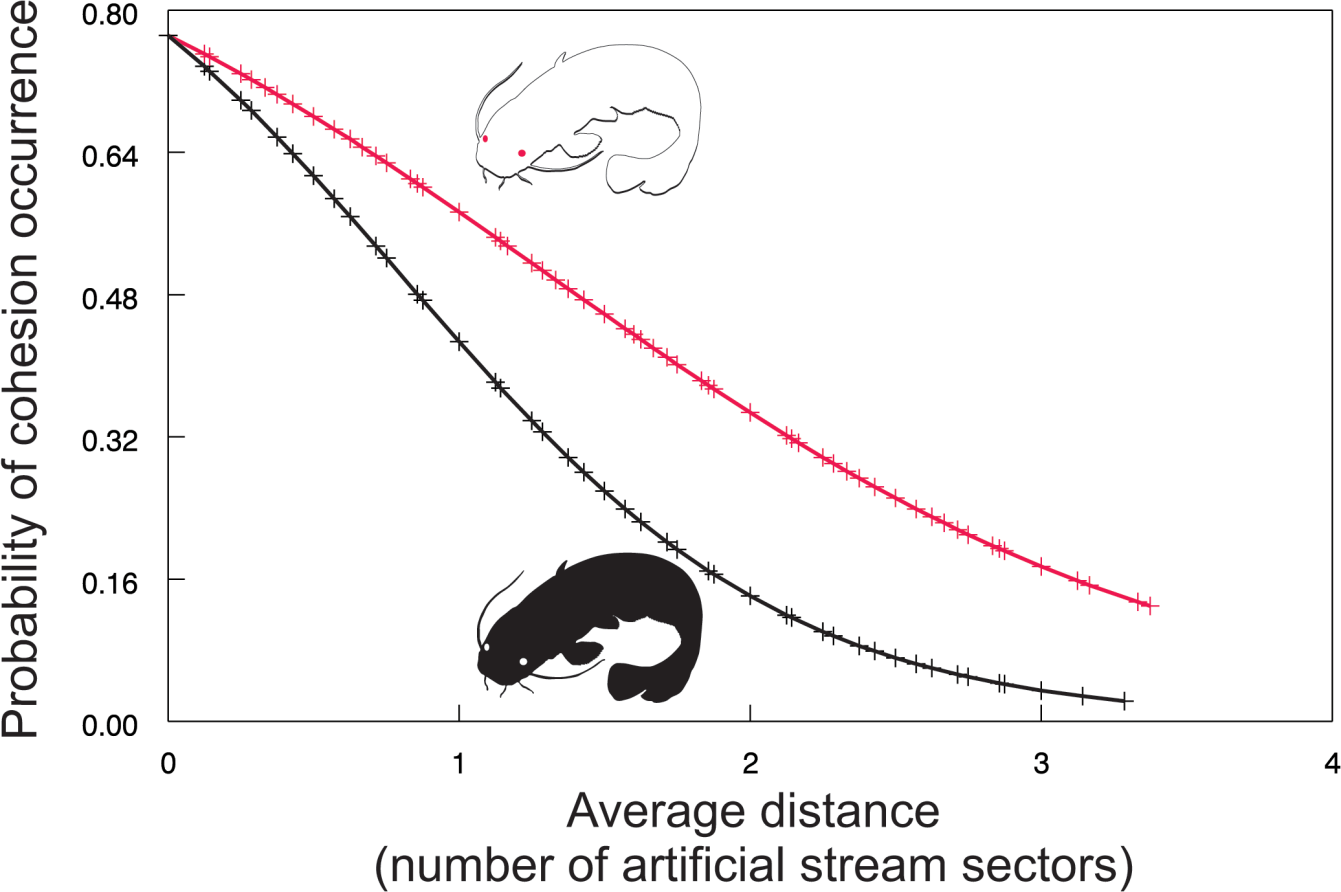
Probability of cohesion occurrence as a function of the average distance between unfamiliar catfish and members of a familiar group. Red line = an albino unfamiliar conspecific; Black line = a pigmented unfamiliar conspecific.

The relative counts of individual isolation and familiar group cohesion occurrence also evolved over time (Fig 4A and 4B). Individual isolation occurrence fluctuated, suggesting gradual decline in the case of albino catfish and relatively stable values in the case of pigmented conspecific. However, group cohesion showed a similar trend for both treatments, suggesting an opposite pattern in contrast to the average distance of an unfamiliar individual from the group (Figs 4B and 2). Further analyses supported this suggestion as the probability of familiar group cohesion occurrence increased with decreasing average distance between the group and unfamiliar conspecific (χ^2^ = 16.20, P<0.0003; Fig 5). In other words, the closer the unfamiliar conspecific was, the more cohesive was the group, with a stronger response in the case of albino catfish (χ^2^ = 16.20, d.f. = 2, P<0.0003; Fig 5). In summary, our results show that an albino catfish was ostracised by a group of familiar pigmented conspecifics, suggesting cohesion as a possible strategy for avoiding the former.

## Discussion

Fish organize themselves with similar phenotype individuals [22], for example by a colour [23,24,25]. During our experiment, albino fish did not associate with pigmented conspecifics, as is usual for juvenile catfish [26]. Accordingly, rare albinos in the wild have a social disadvantage. It can be assumed that in direct contact with normally pigmented individuals social communication of albinos is limited, e.g. submissive (albino) conspecifics cannot darken to avoid attacks of dominant conspecifics, as showed for salmonids [27,28]. Rare occurrence of albinos in the wild can be related with disability to use mimicry that maximizes fish fitness [29], which improves the avoidance of predation risk [30]. Conversely, pale colour in the wild is associated with aggressiveness [31] delivering more antagonistic interactions for albino individuals. For example, blind albino Mexican cave tetra (*Astyanax mexicanus*) showed higher aggression when compared with pigmented conspecifics that see [5].

Ostracism is a response of conspecifics to the presence of an individual who differs for various reasons from the expectations of the group [32]. Animals are usually ostracised due to an illness or other behavioural deviation that might threaten the survival of the group. While the group is safe, the ostracised individuals may suffer from the lack of group benefits and can face an early death [33]. Therefore, the main cause of ostracism in albino catfish is the irregularity within the group. The albino individual is a guiding target not only for itself but also for the whole group through the oddity effect, which enhances the success of attacks by predators [34]. The presence of an individual of a different colour also reduces the effectiveness of the “confusion effect”—when prey migrates toward a shoal to attenuate the singling out and targeting of predatory attacks [35]. Hence, the reason for the exclusion of an individual is not directly due to the difference itself but rather for a fear of oddity. Both, “oddity and confusion effects” are included in a postulate stating that the high visibility of albinos results in their rare occurrence in the wild [3]. Solitary occurrence, demonstrated by the aforementioned ostracism in albino catfish, significantly increases the speed of prey capture by a predator [35].

The average distance of the tested individuals and the fish group evolved over time (Fig 2). The approaching position of both unfamiliar individuals (either albino or pigmented fish) towards a socially established group of fish was most likely the result of the 24 h repeated contact of the fish in the spatially limited laboratory conditions. Fish can learn as proved e.g. to have a colour preference [36]. In open space, the pigmented individuals will most likely discard the albinos at first contact. As indicated by Fig 2, the albino was furthest from the fish group when compared with the pigmented fish at time zero. The psychological, social, and personal aspects of albinism appear to be underestimated. Therefore, further research is needed to better understand other issues associated with albinism [12].

## Supporting Information

S1 Table Data entering the statistical analyses. (PDF)
