# Supplementary material for: Ostracism of an Albino Individual by a Group of Pigmented Catfish
Source: PLoS One. 2015 May 27;10(5):e0128279. doi: 10.1371/journal.pone.0128279 (PMC4446300; doi:10.1371/journal.pone.0128279)
Supplement: S1 Table — (PDF) [file pone.0128279.s001.pdf]

ID	timeSec	Albino	AVG_distance	cohesion	isolation
1	600	1	1.6	1	1
1	1200	1	2	1	1
1	1800	1	0.333	1	0
1	2400	1	0.333	1	0
1	3000	1	0.5	1	0
1	3600	1	0.833	1	0
1	4200	1	1.833	1	1
1	4800	1	1.833	1	1
1	5400	1	1.833	1	1
1	6000	1	1.833	1	1
1	6600	1	1.833	1	1
1	7200	1	1.833	1	1
1	7800	1	2.167	1	1
1	8400	1	2.333	1	1
1	9000	1	2.333	1	1
1	9600	1	1.833	0	0
1	10200	1	2.333	1	1
1	10800	1	2.5	1	1
1	11400	1	2.333	1	1
1	12000	1	2.333	1	1
1	12600	1	2	1	1
1	13200	1	1.833	1	1
1	13800	1	1.833	1	1
1	14400	1	1.667	0	0
1	15000	1	1.5	0	0
1	15600	1	1.667	0	0
1	16200	1	1.333	0	0
1	16800	1	1.167	0	0
1	17400	1	1.833	0	0
1	18000	1	1.833	0	0
1	18600	1	1.833	0	0
1	19200	1	2	1	1
1	19800	1	2	1	1
1	20400	1	2	1	1
1	21000	1	2	1	1
1	21600	1	2.167	1	1
1	22200	1	2.333	0	1
1	22800	1	2.333	0	1
1	23400	1	1.833	0	0
1	24000	1	1.833	0	0
1	24600	1	2.167	0	0
1	25200	1	1.833	0	0
1	25800	1	2.333	0	0
1	26400	1	1.5	0	0
1	27000	1	1.5	0	0
1	27600	1	2.167	0	0
1	28200	1	2.167	0	0

1	28800	1	2.667	0	1
1	29400	1	2.5	0	1
1	30000	1	1	1	0
1	30600	1	2.833	0	1
1	31200	1	2.333	0	0
1	31800	1	2.333	1	1
1	32400	1	3.333	1	1
1	33000	1	2.833	0	1
1	33600	1	2.5	0	1
1	34200	1	2.167	0	1
1	34800	1	2.5	0	0
1	35400	1	2.833	0	0
1	36000	1	1.333	0	0
1	36600	1	2.333	0	1
1	37200	1	3.167	1	1
1	37800	1	2.333	0	0
1	38400	1	2.167	0	0
1	39000	1	1.5	0	0
1	39600	1	1.167	0	0
1	40200	1	2.333	0	1
1	40800	1	1.833	0	0
1	41400	1	1.667	0	0
1	42000	1	1.667	0	0
1	42600	1	1.333	0	0
1	43200	1	1.5	0	0
1	43800	1	1.167	1	0
1	44400	1	1.167	1	0
1	45000	1	1.167	1	0
1	45600	1	1.5	1	0
1	46200	1	1.667	0	0
1	46800	1	1.833	1	1
1	47400	1	1.5	1	1
1	48000	1	1.167	1	0
1	48600	1	1.167	1	0
1	49200	1	1.167	1	0
1	49800	1	1.167	1	0
1	50400	1	2	1	1
1	51000	1	1.5	0	0
1	51600	1	0.833	0	0
1	52200	1	1.333	0	0
1	52800	1	1.333	1	0
1	53400	1	1.333	1	0
1	54000	1	1	1	0
1	54600	1	1.167	1	0
1	55200	1	1	1	0
1	55800	1	1	1	0
1	56400	1	1.333	0	0
1	57000	1	1.833	1	1

1	57600	1	1	1	0
1	58200	1	1.667	0	1
1	58800	1	1.333	0	0
1	59400	1	1.833	0	1
1	60000	1	1.167	0	0
1	60600	1	1.167	1	0
1	61200	1	1.667	0	0
1	61800	1	1.167	1	0
1	62400	1	2	1	1
1	63000	1	1.333	0	0
1	63600	1	1.333	1	0
1	64200	1	1	1	0
1	64800	1	1	1	0
1	65400	1	1.167	0	0
1	66000	1	1.5	1	0
1	66600	1	0.667	1	0
1	67200	1	1	1	0
1	67800	1	1.333	1	1
1	68400	1	1.167	0	0
1	69000	1	1.333	1	0
1	69600	1	1.167	0	0
1	70200	1	1.167	0	0
1	70800	1	1.167	1	0
1	71400	1	1.5	0	0
1	72000	1	1.833	0	0
1	72600	1	1.167	0	0
1	73200	1	0.5	1	0
1	73800	1	0.5	1	0
1	74400	1	0.667	1	0
1	75000	1	0.667	1	0
1	75600	1	1.5	1	1
1	76200	1	0.833	1	0
1	76800	1	0.667	1	0
1	77400	1	0.833	1	0
1	78000	1	0.833	1	0
1	78600	1	1	1	0
1	79200	1	1	1	0
1	79800	1	1	1	0
1	80400	1	1	1	0
1	81000	1	1	1	0
1	81600	1	1	1	0
1	82200	1	1	1	0
1	82800	1	1	1	0
1	83400	1	1	1	0
1	84000	1	1	1	0
1	84600	1	1	1	0
1	85200	1	1	1	0
1	85800	1	1	1	0

1	86400	1	1	1	0
2	600	1	0.875	1	0
2	1200	1	1	0	0
2	1800	1	2.125	0	1
2	2400	1	1.125	1	1
2	3000	1	2.125	0	1
2	3600	1	1	1	0
2	4200	1	0.875	0	0
2	4800	1	1	0	0
2	5400	1	0.875	1	0
2	6000	1	1.75	1	1
2	6600	1	1.875	1	1
2	7200	1	1.125	0	0
2	7800	1	2	1	1
2	8400	1	1.625	1	1
2	9000	1	1.75	0	0
2	9600	1	0.875	1	0
2	10200	1	0.375	1	0
2	10800	1	0.375	1	0
2	11400	1	1.25	1	0
2	12000	1	0.5	1	0
2	12600	1	1.25	1	0
2	13200	1	2.125	1	1
2	13800	1	2.25	1	1
2	14400	1	1	1	0
2	15000	1	1	1	0
2	15600	1	1	1	0
2	16200	1	1	1	0
2	16800	1	1	1	0
2	17400	1	2.25	0	1
2	18000	1	2	1	1
2	18600	1	0.25	1	0
2	19200	1	0.375	1	0
2	19800	1	0.625	0	0
2	20400	1	0.75	1	0
2	21000	1	0.375	1	0
2	21600	1	0.875	1	0
2	22200	1	0.375	1	0
2	22800	1	0.25	1	0
2	23400	1	0.5	1	0
2	24000	1	1	1	0
2	24600	1	1.125	1	0
2	25200	1	1.25	1	0
2	25800	1	2.125	1	1
2	26400	1	1.625	0	0
2	27000	1	1.625	0	0
2	27600	1	2.5	0	1
2	28200	1	2	1	1

2	28800	1	1.875	1	1
2	29400	1	2.375	1	1
2	30000	1	2	1	1
2	30600	1	1.875	1	1
2	31200	1	1.875	1	1
2	31800	1	1	0	0
2	32400	1	1.125	0	0
2	33000	1	1.75	0	0
2	33600	1	1.875	0	0
2	34200	1	0.625	1	0
2	34800	1	1.75	0	0
2	35400	1	1.875	1	1
2	36000	1	1.625	0	0
2	36600	1	0.75	0	0
2	37200	1	1.625	0	1
2	37800	1	2.25	0	1
2	38400	1	1.375	0	0
2	39000	1	1.25	0	0
2	39600	1	0.875	0	0
2	40200	1	0.75	1	0
2	40800	1	0.625	1	0
2	41400	1	0.875	0	0
2	42000	1	1.5	0	0
2	42600	1	0.875	0	0
2	43200	1	2	0	0
2	43800	1	0.75	0	0
2	44400	1	1.875	1	1
2	45000	1	1.25	0	1
2	45600	1	0.875	0	0
2	46200	1	1	1	0
2	46800	1	0.5	0	0
2	47400	1	1.375	0	0
2	48000	1	1.25	1	0
2	48600	1	1	1	0
2	49200	1	1.5	0	0
2	49800	1	0.875	1	0
2	50400	1	1.125	0	0
2	51000	1	1.875	0	0
2	51600	1	1.625	0	0
2	52200	1	1.875	0	0
2	52800	1	2.125	0	0
2	53400	1	1.125	1	0
2	54000	1	2.5	0	1
2	54600	1	2.125	1	1
2	55200	1	0.625	0	0
2	55800	1	2	0	0
2	56400	1	1.25	0	0
2	57000	1	1.75	1	1

2	57600	1	1	0	0
2	58200	1	0.875	0	0
2	58800	1	0.875	0	0
2	59400	1	1.125	0	0
2	60000	1	1.375	0	0
2	60600	1	2.5	0	1
2	61200	1	0.875	1	0
2	61800	1	0.875	0	0
2	62400	1	1.875	0	0
2	63000	1	0.875	0	0
2	63600	1	1	0	0
2	64200	1	2.125	0	0
2	64800	1	1.625	0	0
2	65400	1	1.75	0	1
2	66000	1	1.125	0	0
2	66600	1	2	0	0
2	67200	1	2.75	0	0
2	67800	1	1.125	0	0
2	68400	1	1	1	0
2	69000	1	1.375	1	0
2	69600	1	1.125	1	0
2	70200	1	1.125	1	0
2	70800	1	1.625	0	0
2	71400	1	2	0	0
2	72000	1	1.125	0	0
2	72600	1	0.625	0	0
2	73200	1	0.75	0	0
2	73800	1	1.5	0	0
2	74400	1	0.625	0	0
2	75000	1	1	0	0
2	75600	1	1.375	0	0
2	76200	1	1.25	1	0
2	76800	1	1.75	0	1
2	77400	1	0.875	0	0
2	78000	1	1.875	0	1
2	78600	1	1.875	1	1
2	79200	1	0.875	0	0
2	79800	1	1.75	0	0
2	80400	1	2.125	0	1
2	81000	1	1	1	0
2	81600	1	2.125	0	0
2	82200	1	1	0	0
2	82800	1	1.75	0	1
2	83400	1	0.75	0	0
2	84000	1	1	0	0
2	84600	1	1	0	0
2	85200	1	1	0	0
2	85800	1	2.125	0	0

2	86400	1	1.75	0	0
3	600	1	3	1	1
3	1200	1	0.333	1	0
3	1800	1	1	1	1
3	2400	1	1	1	1
3	3000	1	1	1	1
3	3600	1	0	1	0
3	4200	1	1	1	1
3	4800	1	1.167	1	1
3	5400	1	0	1	0
3	6000	1	1	1	1
3	6600	1	0.333	1	0
3	7200	1	1.714	1	0
3	7800	1	0.286	1	0
3	8400	1	0.286	1	0
3	9000	1	0.286	1	0
3	9600	1	0.286	1	0
3	10200	1	0.571	1	0
3	10800	1	0.429	1	0
3	11400	1	0.286	1	0
3	12000	1	1	1	0
3	12600	1	2.143	1	1
3	13200	1	0.857	1	0
3	13800	1	1.571	1	1
3	14400	1	1.571	1	1
3	15000	1	1.571	1	1
3	15600	1	0.571	1	0
3	16200	1	0.571	1	0
3	16800	1	0.571	1	0
3	17400	1	1.286	0	0
3	18000	1	0.429	1	0
3	18600	1	0.429	1	0
3	19200	1	0.571	1	0
3	19800	1	0.286	1	0
3	20400	1	0.286	1	0
3	21000	1	0.714	1	0
3	21600	1	0.286	1	0
3	22200	1	1.143	0	0
3	22800	1	1.714	1	1
3	23400	1	0.571	1	0
3	24000	1	1.286	0	0
3	24600	1	1.143	0	0
3	25200	1	1	0	0
3	25800	1	0.714	1	0
3	26400	1	0.714	1	0
3	27000	1	0.857	0	0
3	27600	1	1.143	1	0
3	28200	1	1	1	0

3	28800	1	0.857	1	0
3	29400	1	0.857	1	0
3	30000	1	0.857	1	0
3	30600	1	0.857	1	0
3	31200	1	2.714	1	1
3	31800	1	1.286	1	0
3	32400	1	0.857	0	0
3	33000	1	1.286	0	0
3	33600	1	1.286	0	0
3	34200	1	1.286	0	0
3	34800	1	1.857	0	0
3	35400	1	2.143	0	1
3	36000	1	1.143	0	0
3	36600	1	0.857	0	0
3	37200	1	0.571	1	0
3	37800	1	0.571	1	0
3	38400	1	1.857	1	1
3	39000	1	1.143	0	0
3	39600	1	1.429	0	0
3	40200	1	0.857	0	0
3	40800	1	1.571	0	1
3	41400	1	1	0	0
3	42000	1	2.143	0	0
3	42600	1	1.143	0	0
3	43200	1	0.714	0	0
3	43800	1	0.714	0	0
3	44400	1	1.286	0	0
3	45000	1	1.714	0	1
3	45600	1	0.857	1	0
3	46200	1	1.429	0	0
3	46800	1	1.143	0	0
3	47400	1	1	0	0
3	48000	1	1.429	0	0
3	48600	1	1	0	0
3	49200	1	1.857	0	0
3	49800	1	1.571	0	0
3	50400	1	1.143	0	0
3	51000	1	1.286	0	0
3	51600	1	1.714	1	1
3	52200	1	1.143	0	0
3	52800	1	1.429	0	1
3	53400	1	1.143	0	0
3	54000	1	0.714	0	0
3	54600	1	0.857	0	0
3	55200	1	1.286	0	1
3	55800	1	0.857	0	0
3	56400	1	0.714	0	0
3	57000	1	0.857	1	0

3	57600	1	1.143	0	0
3	58200	1	0.857	0	0
3	58800	1	0.857	0	0
3	59400	1	1.143	0	0
3	60000	1	0.714	0	0
3	60600	1	1.571	1	1
3	61200	1	1.857	1	1
3	61800	1	1.286	1	0
3	62400	1	1.714	1	1
3	63000	1	0.571	0	0
3	63600	1	2	1	1
3	64200	1	1.857	0	0
3	64800	1	1.143	0	0
3	65400	1	1	1	1
3	66000	1	2.429	1	1
3	66600	1	3	1	1
3	67200	1	2.571	0	0
3	67800	1	1.714	0	0
3	68400	1	2	0	1
3	69000	1	2.143	0	0
3	69600	1	1.571	0	0
3	70200	1	1.286	0	1
3	70800	1	2.429	0	1
3	71400	1	1.286	0	0
3	72000	1	1	0	0
3	72600	1	1.857	0	0
3	73200	1	2	0	0
3	73800	1	1.571	0	0
3	74400	1	1	0	0
3	75000	1	1.857	0	0
3	75600	1	1.286	0	1
3	76200	1	0.857	0	0
3	76800	1	2.143	0	1
3	77400	1	1	0	0
3	78000	1	1	0	0
3	78600	1	0.857	0	0
3	79200	1	2	1	1
3	79800	1	0.857	1	0
3	80400	1	1.286	1	0
3	81000	1	1.286	1	0
3	81600	1	1.286	1	0
3	82200	1	0.429	1	0
3	82800	1	2.714	1	1
3	83400	1	2.714	1	1
3	84000	1	2.714	1	1
3	84600	1	2.714	1	1
3	85200	1	2.714	1	1
3	85800	1	2.714	1	1

3	86400	1	2.714	1	1
4	600	1	.	1	0
4	1200	1	.	1	0
4	1800	1	0.429	1	0
4	2400	1	0.571	1	0
4	3000	1	0.429	1	0
4	3600	1	0.429	1	0
4	4200	1	0.571	1	0
4	4800	1	0.571	1	0
4	5400	1	0.571	1	0
4	6000	1	0.571	1	0
4	6600	1	0.571	1	0
4	7200	1	0.571	1	0
4	7800	1	0.571	1	0
4	8400	1	0.571	1	0
4	9000	1	0.571	1	0
4	9600	1	0.571	1	0
4	10200	1	0.571	1	0
4	10800	1	0.571	1	0
4	11400	1	0.571	1	0
4	12000	1	0.429	1	0
4	12600	1	0.571	0	0
4	13200	1	0.571	0	0
4	13800	1	0.143	1	0
4	14400	1	0.143	1	0
4	15000	1	0.429	0	0
4	15600	1	0.143	1	0
4	16200	1	0.857	1	0
4	16800	1	0.857	1	0
4	17400	1	0.429	0	0
4	18000	1	0.143	1	0
4	18600	1	0.143	1	0
4	19200	1	0.143	1	0
4	19800	1	0.143	1	0
4	20400	1	0.143	1	0
4	21000	1	0.429	1	0
4	21600	1	0.286	1	0
4	22200	1	0.429	1	0
4	22800	1	0.857	1	0
4	23400	1	0.286	1	0
4	24000	1	0.286	1	0
4	24600	1	0.714	1	0
4	25200	1	0.571	1	0
4	25800	1	0.286	1	0
4	26400	1	0.429	1	0
4	27000	1	0.714	0	0
4	27600	1	1	0	0
4	28200	1	2	0	0

4	28800	1	1.429	0	0
4	29400	1	1.714	0	1
4	30000	1	1.429	0	0
4	30600	1	1	1	0
4	31200	1	1.857	0	0
4	31800	1	0.714	0	0
4	32400	1	1.286	0	0
4	33000	1	2.143	0	0
4	33600	1	1	0	0
4	34200	1	1	1	0
4	34800	1	1.857	0	0
4	35400	1	2.143	0	0
4	36000	1	1.571	0	0
4	36600	1	1	1	0
4	37200	1	1	0	0
4	37800	1	1	0	0
4	38400	1	0.714	1	0
4	39000	1	1.714	1	1
4	39600	1	0.857	0	0
4	40200	1	1.571	1	1
4	40800	1	2	0	0
4	41400	1	2	1	1
4	42000	1	1.571	0	0
4	42600	1	1.286	1	0
4	43200	1	1.857	0	0
4	43800	1	0.714	1	0
4	44400	1	0.857	1	0
4	45000	1	0.857	1	0
4	45600	1	0.429	1	0
4	46200	1	2.429	0	0
4	46800	1	1.857	0	0
4	47400	1	1.286	0	1
4	48000	1	1.857	0	1
4	48600	1	1.714	1	1
4	49200	1	1.429	0	0
4	49800	1	1.429	1	0
4	50400	1	2.714	0	1
4	51000	1	1.857	0	0
4	51600	1	1	0	0
4	52200	1	2.857	1	1
4	52800	1	0.857	0	0
4	53400	1	1.429	0	0
4	54000	1	1.429	0	0
4	54600	1	2.429	0	1
4	55200	1	1.143	0	0
4	55800	1	2.571	0	1
4	56400	1	2.286	0	1
4	57000	1	1.143	0	0

4	57600	1	1	1	0
4	58200	1	1	0	0
4	58800	1	0.857	1	0
4	59400	1	0.714	0	0
4	60000	1	1.571	0	0
4	60600	1	1.286	0	0
4	61200	1	2.143	0	1
4	61800	1	1.571	0	0
4	62400	1	2.143	1	1
4	63000	1	0.857	0	0
4	63600	1	2	0	0
4	64200	1	1.857	0	0
4	64800	1	2	0	0
4	65400	1	1.429	0	0
4	66000	1	1.857	0	0
4	66600	1	1.571	0	0
4	67200	1	0.857	0	0
4	67800	1	1.143	0	0
4	68400	1	0.714	0	0
4	69000	1	0.429	1	0
4	69600	1	1.571	0	0
4	70200	1	0.571	1	0
4	70800	1	1.714	1	1
4	71400	1	1.571	1	1
4	72000	1	1	0	0
4	72600	1	0.714	1	0
4	73200	1	1	1	0
4	73800	1	1	1	1
4	74400	1	0.571	1	0
4	75000	1	1.429	0	0
4	75600	1	1.286	1	0
4	76200	1	0.857	1	0
4	76800	1	1.857	1	1
4	77400	1	2	1	1
4	78000	1	1.857	0	0
4	78600	1	1.286	1	0
4	79200	1	1.571	0	0
4	79800	1	0.571	1	0
4	80400	1	0.571	1	0
4	81000	1	0.857	1	0
4	81600	1	1.429	1	1
4	82200	1	0.571	0	0
4	82800	1	0.714	1	0
4	83400	1	0.857	1	0
4	84000	1	0.857	0	0
4	84600	1	0.857	1	0
4	85200	1	0.571	1	0
4	85800	1	0.714	1	0

4	86400	1	0.857	1	0
5	600	1	.	1	0
5	1200	1	1.857	0	1
5	1800	1	1.857	0	1
5	2400	1	1.857	0	1
5	3000	1	0.286	1	0
5	3600	1	0.286	1	0
5	4200	1	0.714	0	0
5	4800	1	0.429	1	0
5	5400	1	0.429	1	0
5	6000	1	0.429	1	0
5	6600	1	0.429	1	0
5	7200	1	0.429	1	0
5	7800	1	0.429	1	0
5	8400	1	0.429	1	0
5	9000	1	0.429	1	0
5	9600	1	0.429	1	0
5	10200	1	0.429	1	0
5	10800	1	0.429	1	0
5	11400	1	0.571	1	0
5	12000	1	0.571	1	0
5	12600	1	0.571	1	0
5	13200	1	0.571	1	0
5	13800	1	0.571	1	0
5	14400	1	0.571	1	0
5	15000	1	0.571	1	0
5	15600	1	0.714	1	0
5	16200	1	0.5	1	0
5	16800	1	0.5	1	0
5	17400	1	0.5	1	0
5	18000	1	0.5	1	0
5	18600	1	0.5	1	0
5	19200	1	0.5	1	0
5	19800	1	1	1	0
5	20400	1	1.75	1	1
5	21000	1	0.75	1	0
5	21600	1	1.5	0	0
5	22200	1	1	1	0
5	22800	1	1.75	1	1
5	23400	1	1.875	1	1
5	24000	1	1	1	0
5	24600	1	1.5	0	0
5	25200	1	0.75	1	0
5	25800	1	1.125	0	0
5	26400	1	1.625	1	1
5	27000	1	0.875	0	0
5	27600	1	2.5	1	1
5	28200	1	2.375	0	1

5	28800	1	0.875	0	0
5	29400	1	1	1	0
5	30000	1	3	1	1
5	30600	1	0.875	0	0
5	31200	1	1.125	0	0
5	31800	1	1.5	0	0
5	32400	1	3.125	0	1
5	33000	1	2.25	0	1
5	33600	1	2.125	0	1
5	34200	1	1.25	0	0
5	34800	1	1.125	0	0
5	35400	1	1.5	0	0
5	36000	1	1.75	0	0
5	36600	1	1.25	0	0
5	37200	1	1.75	0	0
5	37800	1	1.875	0	0
5	38400	1	1.75	0	1
5	39000	1	1	0	0
5	39600	1	1.5	0	0
5	40200	1	0.75	0	0
5	40800	1	2.625	0	0
5	41400	1	2	0	0
5	42000	1	1.125	0	0
5	42600	1	2.25	0	0
5	43200	1	1.375	0	1
5	43800	1	2.125	0	0
5	44400	1	2.75	0	0
5	45000	1	2	0	0
5	45600	1	1.5	1	0
5	46200	1	1.25	0	0
5	46800	1	1.625	0	0
5	47400	1	1	0	0
5	48000	1	0.625	1	0
5	48600	1	1	1	0
5	49200	1	2.25	0	1
5	49800	1	0.875	0	0
5	50400	1	1.25	0	0
5	51000	1	1	0	0
5	51600	1	1.125	0	0
5	52200	1	1	0	0
5	52800	1	1.375	0	0
5	53400	1	2.625	0	0
5	54000	1	1	0	0
5	54600	1	1.125	0	0
5	55200	1	1	0	0
5	55800	1	2.5	0	1
5	56400	1	1.25	0	0
5	57000	1	1.625	1	1

5	57600	1	2.5	1	1
5	58200	1	1.125	0	0
5	58800	1	1.25	0	0
5	59400	1	1.875	0	0
5	60000	1	1	0	0
5	60600	1	2.125	1	1
5	61200	1	1.75	0	0
5	61800	1	1.75	0	1
5	62400	1	0.875	0	0
5	63000	1	2.25	0	0
5	63600	1	1	0	0
5	64200	1	0.875	1	0
5	64800	1	1.75	0	0
5	65400	1	0.875	0	0
5	66000	1	1.625	0	0
5	66600	1	2.125	1	1
5	67200	1	0.25	1	0
5	67800	1	1	0	0
5	68400	1	1.625	0	0
5	69000	1	2.25	0	1
5	69600	1	1.875	1	1
5	70200	1	2.25	0	0
5	70800	1	0.875	0	0
5	71400	1	1.5	0	0
5	72000	1	0.875	0	0
5	72600	1	1.375	0	0
5	73200	1	0.625	0	0
5	73800	1	0.625	0	0
5	74400	1	2.125	0	1
5	75000	1	0.75	0	0
5	75600	1	1.875	0	0
5	76200	1	0.625	0	0
5	76800	1	1.5	1	1
5	77400	1	0.625	0	0
5	78000	1	2	0	0
5	78600	1	1.125	0	0
5	79200	1	0.375	1	0
5	79800	1	0.75	1	0
5	80400	1	2.25	1	1
5	81000	1	2.25	1	1
5	81600	1	2.25	1	1
5	82200	1	2.25	1	1
5	82800	1	2.25	1	1
5	83400	1	2.25	1	1
5	84000	1	2.25	1	1
5	84600	1	2.25	1	1
5	85200	1	2.25	1	1
5	85800	1	2.25	1	1

5	86400	1	2.25	1	1
6	600	0	.	1	0
6	1200	0	1.375	1	0
6	1800	0	0.875	1	0
6	2400	0	0.75	1	0
6	3000	0	0.625	1	0
6	3600	0	1.375	1	0
6	4200	0	1.375	1	0
6	4800	0	2.125	1	1
6	5400	0	0.625	1	0
6	6000	0	1.75	0	1
6	6600	0	0.75	1	0
6	7200	0	0.625	1	0
6	7800	0	1.125	1	0
6	8400	0	0.75	0	0
6	9000	0	1.625	0	0
6	9600	0	0.75	1	0
6	10200	0	0.875	0	0
6	10800	0	0.625	0	0
6	11400	0	0.75	1	0
6	12000	0	1.125	0	0
6	12600	0	0.875	0	0
6	13200	0	0.625	1	0
6	13800	0	1	1	0
6	14400	0	1.875	0	0
6	15000	0	2.25	1	1
6	15600	0	1	1	0
6	16200	0	1.125	1	0
6	16800	0	0.625	1	0
6	17400	0	0.625	1	0
6	18000	0	0.625	1	0
6	18600	0	0.625	1	0
6	19200	0	0.75	1	0
6	19800	0	0.75	1	0
6	20400	0	0.625	1	0
6	21000	0	0.625	1	0
6	21600	0	0.75	1	0
6	22200	0	1.375	1	0
6	22800	0	1.125	1	0
6	23400	0	2.125	0	0
6	24000	0	1.375	0	0
6	24600	0	1.5	0	0
6	25200	0	1.5	0	0
6	25800	0	1.125	0	0
6	26400	0	2.375	0	1
6	27000	0	2.25	0	0
6	27600	0	1.625	0	0
6	28200	0	1.25	0	0

6	28800	0	0.875	0	0
6	29400	0	1.625	1	0
6	30000	0	2.75	1	1
6	30600	0	1.25	0	1
6	31200	0	2.25	0	1
6	31800	0	2.75	0	0
6	32400	0	1.5	0	0
6	33000	0	1.625	0	0
6	33600	0	1	0	0
6	34200	0	1.75	0	0
6	34800	0	1.75	0	0
6	35400	0	0.75	1	0
6	36000	0	1.5	0	0
6	36600	0	1.875	0	0
6	37200	0	2.375	0	1
6	37800	0	2.5	0	1
6	38400	0	1.75	0	0
6	39000	0	2.125	0	1
6	39600	0	1.5	0	0
6	40200	0	0.75	1	0
6	40800	0	2.75	1	1
6	41400	0	1.5	0	0
6	42000	0	1.25	0	0
6	42600	0	0.625	0	0
6	43200	0	1	0	0
6	43800	0	2.375	0	0
6	44400	0	1.25	0	0
6	45000	0	1	0	0
6	45600	0	1.125	1	0
6	46200	0	1.375	1	1
6	46800	0	1.125	0	0
6	47400	0	1.375	0	0
6	48000	0	0.75	0	0
6	48600	0	1.125	0	0
6	49200	0	1.875	0	0
6	49800	0	1.625	1	1
6	50400	0	1.5	0	0
6	51000	0	0.75	0	0
6	51600	0	1.75	0	0
6	52200	0	0.75	0	0
6	52800	0	1.375	0	0
6	53400	0	2	0	0
6	54000	0	1.875	0	1
6	54600	0	1.25	0	0
6	55200	0	1.75	0	0
6	55800	0	1.125	0	0
6	56400	0	1	0	0
6	57000	0	1.625	0	0

6	57600	0	1	0	0
6	58200	0	1	0	0
6	58800	0	2.25	0	0
6	59400	0	0.875	0	0
6	60000	0	1.25	0	0
6	60600	0	1.25	0	0
6	61200	0	0.875	0	0
6	61800	0	2.25	0	0
6	62400	0	1.75	1	1
6	63000	0	1.375	0	0
6	63600	0	1.5	0	0
6	64200	0	1	0	0
6	64800	0	0.75	0	0
6	65400	0	1	0	0
6	66000	0	0.75	0	0
6	66600	0	1.5	0	1
6	67200	0	1.25	0	0
6	67800	0	1	0	0
6	68400	0	0.625	0	0
6	69000	0	1.25	0	0
6	69600	0	0.5	1	0
6	70200	0	1.25	0	0
6	70800	0	1.125	0	0
6	71400	0	0.875	1	0
6	72000	0	1.875	0	0
6	72600	0	0.625	1	0
6	73200	0	1.5	0	0
6	73800	0	1	1	0
6	74400	0	0.875	0	0
6	75000	0	0.875	0	0
6	75600	0	0.875	0	0
6	76200	0	1	0	0
6	76800	0	0.875	0	0
6	77400	0	1.375	0	1
6	78000	0	0.875	0	0
6	78600	0	0.875	0	0
6	79200	0	1.125	0	0
6	79800	0	0.875	0	0
6	80400	0	0.875	1	0
6	81000	0	1.375	0	0
6	81600	0	1.875	1	1
6	82200	0	0.5	1	0
6	82800	0	1.5	1	0
6	83400	0	0.25	1	0
6	84000	0	0.875	1	0
6	84600	0	0.875	1	0
6	85200	0	0.375	1	0
6	85800	0	1	1	0

6	86400	0	0.625	1	0
7	600	0	0.5	0	0
7	1200	0	0.125	1	0
7	1800	0	0.5	0	0
7	2400	0	0.5	0	0
7	3000	0	0.5	0	0
7	3600	0	0.5	0	0
7	4200	0	0.875	0	0
7	4800	0	0.125	1	0
7	5400	0	0.25	1	0
7	6000	0	0.25	1	0
7	6600	0	1	1	0
7	7200	0	1.125	1	1
7	7800	0	1.125	0	0
7	8400	0	1.375	0	0
7	9000	0	1.125	1	0
7	9600	0	1	1	0
7	10200	0	0.5	1	0
7	10800	0	0.625	0	0
7	11400	0	1	0	0
7	12000	0	0.75	1	0
7	12600	0	0.875	1	0
7	13200	0	0.875	0	0
7	13800	0	0.625	1	0
7	14400	0	0.875	0	0
7	15000	0	0.5	1	0
7	15600	0	0.75	1	0
7	16200	0	0.5	1	0
7	16800	0	0.75	1	0
7	17400	0	1.375	0	0
7	18000	0	1.375	0	0
7	18600	0	0.625	0	0
7	19200	0	0.625	0	0
7	19800	0	0.625	0	0
7	20400	0	0.875	0	0
7	21000	0	1.375	0	0
7	21600	0	1.375	0	0
7	22200	0	1.5	0	0
7	22800	0	1.625	0	0
7	23400	0	1.5	0	0
7	24000	0	1.5	0	0
7	24600	0	1.5	0	0
7	25200	0	1.5	0	0
7	25800	0	2.875	1	1
7	26400	0	0.875	0	0
7	27000	0	0.75	1	0
7	27600	0	0.625	1	0
7	28200	0	0.75	1	0

7	28800	0	1.125	1	0
7	29400	0	1.625	0	0
7	30000	0	1.25	0	1
7	30600	0	1	1	0
7	31200	0	1	1	0
7	31800	0	1.25	0	0
7	32400	0	3	1	1
7	33000	0	2	0	0
7	33600	0	2.125	0	0
7	34200	0	1.125	0	0
7	34800	0	1.125	1	0
7	35400	0	1.875	0	1
7	36000	0	1.5	0	0
7	36600	0	1.25	0	0
7	37200	0	1.125	0	0
7	37800	0	1.25	0	0
7	38400	0	1	0	0
7	39000	0	1.5	0	0
7	39600	0	1.5	0	1
7	40200	0	2.25	0	0
7	40800	0	1.25	0	0
7	41400	0	1.25	0	0
7	42000	0	0.875	0	0
7	42600	0	1.125	0	0
7	43200	0	2.25	0	1
7	43800	0	1.375	0	0
7	44400	0	1.25	0	0
7	45000	0	0.75	1	0
7	45600	0	1.75	0	0
7	46200	0	1.25	0	0
7	46800	0	1.125	0	0
7	47400	0	1.5	1	1
7	48000	0	0.625	1	0
7	48600	0	1.25	1	0
7	49200	0	1.625	0	0
7	49800	0	2.125	0	0
7	50400	0	1.375	0	0
7	51000	0	1.5	1	1
7	51600	0	2	0	1
7	52200	0	0.875	0	0
7	52800	0	1	0	0
7	53400	0	0.5	1	0
7	54000	0	1.125	0	0
7	54600	0	0.75	1	0
7	55200	0	0.5	1	0
7	55800	0	0.875	0	0
7	56400	0	0.75	1	0
7	57000	0	1.5	1	0

7	57600	0	1	0	0
7	58200	0	1	0	0
7	58800	0	1.625	0	1
7	59400	0	1	0	0
7	60000	0	0.875	0	0
7	60600	0	1	0	0
7	61200	0	0.75	0	0
7	61800	0	0.625	0	0
7	62400	0	0.625	0	0
7	63000	0	1	0	0
7	63600	0	1.125	0	0
7	64200	0	1.625	0	1
7	64800	0	0.75	0	0
7	65400	0	1	0	0
7	66000	0	0.75	1	0
7	66600	0	1	0	0
7	67200	0	0.875	0	0
7	67800	0	0.75	1	0
7	68400	0	1	1	0
7	69000	0	1	0	0
7	69600	0	0.875	0	0
7	70200	0	0.75	0	0
7	70800	0	1.125	0	0
7	71400	0	0.75	0	0
7	72000	0	0.625	1	0
7	72600	0	0.625	0	0
7	73200	0	1.25	0	0
7	73800	0	0.75	0	0
7	74400	0	0.875	0	0
7	75000	0	0.5	0	0
7	75600	0	1.5	1	0
7	76200	0	0.625	1	0
7	76800	0	0.625	1	0
7	77400	0	0.875	0	0
7	78000	0	0.375	1	0
7	78600	0	0.875	1	0
7	79200	0	0.5	1	0
7	79800	0	0.625	1	0
7	80400	0	0.875	1	0
7	81000	0	0.375	1	0
7	81600	0	0.375	1	0
7	82200	0	0.375	1	0
7	82800	0	0.375	1	0
7	83400	0	0.375	1	0
7	84000	0	0.375	1	0
7	84600	0	0.375	1	0
7	85200	0	0.375	1	0
7	85800	0	0.375	1	0

7	86400	0	0.375	1	0
8	600	0	0.143	1	0
8	1200	0	0.714	1	0
8	1800	0	0.714	1	0
8	2400	0	0.714	1	0
8	3000	0	0.429	1	0
8	3600	0	0.429	1	0
8	4200	0	0.429	1	0
8	4800	0	0.429	1	0
8	5400	0	0.429	1	0
8	6000	0	0.429	1	0
8	6600	0	0.571	1	0
8	7200	0	0.571	1	0
8	7800	0	0.571	1	0
8	8400	0	0.571	1	0
8	9000	0	0.571	1	0
8	9600	0	1	1	0
8	10200	0	0.571	1	0
8	10800	0	0.571	1	0
8	11400	0	0.571	1	0
8	12000	0	0.571	1	0
8	12600	0	0.857	1	0
8	13200	0	1	0	0
8	13800	0	0.286	1	0
8	14400	0	1.143	1	0
8	15000	0	1	1	0
8	15600	0	2	1	1
8	16200	0	0.143	1	0
8	16800	0	0.286	1	0
8	17400	0	0.286	1	0
8	18000	0	0.143	1	0
8	18600	0	0.429	1	0
8	19200	0	0.571	0	0
8	19800	0	0.429	1	0
8	20400	0	0.429	1	0
8	21000	0	1	1	0
8	21600	0	0.286	1	0
8	22200	0	0.714	0	0
8	22800	0	0.857	0	0
8	23400	0	0.571	1	0
8	24000	0	0.714	1	0
8	24600	0	0.571	1	0
8	25200	0	1.571	1	1
8	25800	0	0.571	1	0
8	26400	0	0.714	1	0
8	27000	0	0.571	1	0
8	27600	0	0.857	0	0
8	28200	0	0.571	1	0

8	28800	0	0.857	0	0
8	29400	0	2	0	1
8	30000	0	1.143	1	0
8	30600	0	0.571	1	0
8	31200	0	0.857	1	0
8	31800	0	0.714	1	0
8	32400	0	0.857	0	0
8	33000	0	2.143	0	0
8	33600	0	1.429	1	1
8	34200	0	1	0	0
8	34800	0	1.143	0	0
8	35400	0	0.714	0	0
8	36000	0	2.143	0	1
8	36600	0	1.143	1	0
8	37200	0	1.286	0	1
8	37800	0	1.143	0	0
8	38400	0	0.571	0	0
8	39000	0	2	0	0
8	39600	0	1.143	0	0
8	40200	0	1.429	0	0
8	40800	0	1	0	0
8	41400	0	1.286	0	0
8	42000	0	1.571	0	0
8	42600	0	2.714	0	0
8	43200	0	1.857	0	0
8	43800	0	1.857	0	0
8	44400	0	1.143	0	0
8	45000	0	1.714	0	0
8	45600	0	2.857	0	1
8	46200	0	1.143	0	0
8	46800	0	1.286	0	0
8	47400	0	2.143	0	0
8	48000	0	1.714	0	1
8	48600	0	2	0	0
8	49200	0	1	1	0
8	49800	0	1.429	0	1
8	50400	0	1.143	0	0
8	51000	0	1.143	1	0
8	51600	0	1.857	0	0
8	52200	0	2	0	0
8	52800	0	1.429	0	0
8	53400	0	1.714	0	0
8	54000	0	1.429	0	0
8	54600	0	1.143	0	0
8	55200	0	1.429	0	0
8	55800	0	1.571	0	1
8	56400	0	0.714	1	0
8	57000	0	2.143	0	1

8	57600	0	1.143	0	0
8	58200	0	1.857	0	0
8	58800	0	0.714	1	0
8	59400	0	2.286	1	1
8	60000	0	1.143	0	0
8	60600	0	3.286	1	1
8	61200	0	2.429	1	1
8	61800	0	2.429	0	0
8	62400	0	1.143	1	0
8	63000	0	1.143	0	0
8	63600	0	3	1	1
8	64200	0	2	0	0
8	64800	0	1	1	0
8	65400	0	1	0	0
8	66000	0	1.714	0	1
8	66600	0	1.286	0	0
8	67200	0	1.286	0	0
8	67800	0	1.571	0	0
8	68400	0	1.571	0	0
8	69000	0	1.429	0	0
8	69600	0	2	0	0
8	70200	0	1.571	0	0
8	70800	0	1.571	0	0
8	71400	0	2	0	1
8	72000	0	1.571	0	0
8	72600	0	1.143	0	0
8	73200	0	0.857	0	0
8	73800	0	1.286	0	1
8	74400	0	1.429	0	1
8	75000	0	2.286	0	0
8	75600	0	2.143	0	1
8	76200	0	1.286	0	1
8	76800	0	1.143	0	0
8	77400	0	1.143	0	0
8	78000	0	1.286	0	1
8	78600	0	1	1	0
8	79200	0	1.571	0	0
8	79800	0	1	1	0
8	80400	0	0.714	1	0
8	81000	0	1	1	0
8	81600	0	1.286	1	0
8	82200	0	1.143	1	0
8	82800	0	1.143	1	0
8	83400	0	0.714	1	0
8	84000	0	0.857	0	0
8	84600	0	0.714	1	0
8	85200	0	0.857	1	0
8	85800	0	0.714	1	0

8	86400	0	0.714	1	0
9	600	0	1	1	1
9	1200	0	2	1	1
9	1800	0	0	1	0
9	2400	0	0	1	0
9	3000	0	0.286	1	0
9	3600	0	0.143	1	0
9	4200	0	1.143	1	1
9	4800	0	0.429	0	0
9	5400	0	0.143	1	0
9	6000	0	0.571	0	0
9	6600	0	0.429	1	0
9	7200	0	1.143	0	1
9	7800	0	0.857	1	0
9	8400	0	1.571	0	0
9	9000	0	1.571	1	0
9	9600	0	0.429	1	0
9	10200	0	0.429	1	0
9	10800	0	0.429	1	0
9	11400	0	1	1	0
9	12000	0	0.286	1	0
9	12600	0	0.286	1	0
9	13200	0	0.429	1	0
9	13800	0	1	1	0
9	14400	0	1	1	0
9	15000	0	1	1	0
9	15600	0	1	1	0
9	16200	0	1	1	0
9	16800	0	1	1	0
9	17400	0	1.143	1	0
9	18000	0	0.857	1	0
9	18600	0	0.857	1	0
9	19200	0	1	1	0
9	19800	0	1.714	0	0
9	20400	0	0.286	1	0
9	21000	0	0.714	1	0
9	21600	0	0.714	1	0
9	22200	0	0.714	1	0
9	22800	0	0.429	1	0
9	23400	0	2	1	1
9	24000	0	0.714	1	0
9	24600	0	1	0	0
9	25200	0	1.143	1	0
9	25800	0	0.714	1	0
9	26400	0	1.714	0	0
9	27000	0	1.714	0	0
9	27600	0	1.714	0	0
9	28200	0	1.429	0	0

9	28800	0	1.571	0	0
9	29400	0	1.286	0	0
9	30000	0	1.429	0	0
9	30600	0	1	0	0
9	31200	0	1.286	0	0
9	31800	0	2.571	0	1
9	32400	0	0.571	0	0
9	33000	0	1.571	0	0
9	33600	0	1.286	0	1
9	34200	0	1.143	0	0
9	34800	0	1.286	0	0
9	35400	0	1.857	0	0
9	36000	0	1.143	0	0
9	36600	0	1.714	0	0
9	37200	0	1.143	0	1
9	37800	0	2.286	0	0
9	38400	0	2.143	0	0
9	39000	0	2	0	0
9	39600	0	1.286	0	0
9	40200	0	2.143	0	0
9	40800	0	2	0	1
9	41400	0	1.286	0	0
9	42000	0	2.429	0	0
9	42600	0	2	0	0
9	43200	0	0.857	0	0
9	43800	0	1.429	0	0
9	44400	0	1.571	0	0
9	45000	0	1.714	0	0
9	45600	0	1.857	0	0
9	46200	0	1.143	0	0
9	46800	0	1.143	0	0
9	47400	0	1.571	0	0
9	48000	0	1.143	1	0
9	48600	0	0.714	0	0
9	49200	0	1.286	0	0
9	49800	0	1.143	0	0
9	50400	0	1.143	0	0
9	51000	0	1.857	0	0
9	51600	0	1.571	0	0
9	52200	0	1.143	0	0
9	52800	0	1.286	0	0
9	53400	0	1.143	1	0
9	54000	0	1	0	0
9	54600	0	1.286	0	0
9	55200	0	1.429	0	0
9	55800	0	0.571	1	0
9	56400	0	1.429	0	0
9	57000	0	1.429	0	0

9	57600	0	0.714	1	0
9	58200	0	0.714	0	0
9	58800	0	1.286	1	0
9	59400	0	0.714	1	0
9	60000	0	1.714	0	0
9	60600	0	0.714	1	0
9	61200	0	1.571	0	0
9	61800	0	1.286	0	0
9	62400	0	1.429	0	0
9	63000	0	1.143	0	0
9	63600	0	2	0	0
9	64200	0	1.286	1	0
9	64800	0	2	0	0
9	65400	0	0.857	1	0
9	66000	0	0.714	0	0
9	66600	0	1	0	0
9	67200	0	1.143	0	0
9	67800	0	1.143	0	0
9	68400	0	1.143	0	0
9	69000	0	1.429	0	1
9	69600	0	1.286	1	0
9	70200	0	1	0	0
9	70800	0	0.857	1	0
9	71400	0	1.429	0	0
9	72000	0	1	0	0
9	72600	0	2	0	0
9	73200	0	1.571	0	0
9	73800	0	0.571	0	0
9	74400	0	1.857	1	1
9	75000	0	1.286	0	0
9	75600	0	1.143	0	0
9	76200	0	1.571	0	0
9	76800	0	1.143	1	0
9	77400	0	2.143	0	0
9	78000	0	1.571	0	0
9	78600	0	1	0	0
9	79200	0	1	0	0
9	79800	0	1	0	0
9	80400	0	1	0	0
9	81000	0	1	0	0
9	81600	0	1	0	0
9	82200	0	1	0	0
9	82800	0	1	0	0
9	83400	0	1	0	0
9	84000	0	1	0	0
9	84600	0	1	0	0
9	85200	0	1	0	0
9	85800	0	1	0	0

9	86400	0	1	0	0
10	600	0	1.286	0	1
10	1200	0	0.286	1	0
10	1800	0	0.286	1	0
10	2400	0	0.286	1	0
10	3000	0	0.286	1	0
10	3600	0	0.75	1	0
10	4200	0	0.75	0	0
10	4800	0	0.625	0	0
10	5400	0	0.875	1	0
10	6000	0	1.25	1	0
10	6600	0	0.75	1	0
10	7200	0	0.625	1	0
10	7800	0	1.125	0	1
10	8400	0	0.5	1	0
10	9000	0	0.75	1	0
10	9600	0	0.75	1	0
10	10200	0	2	1	1
10	10800	0	1	0	0
10	11400	0	0.875	0	0
10	12000	0	0.625	0	0
10	12600	0	0.5	1	0
10	13200	0	0.875	1	0
10	13800	0	0.875	1	0
10	14400	0	0.375	1	0
10	15000	0	1.25	1	1
10	15600	0	2.875	1	1
10	16200	0	0.875	1	0
10	16800	0	0.75	1	0
10	17400	0	0.625	1	0
10	18000	0	0.625	1	0
10	18600	0	1.625	1	1
10	19200	0	0.625	1	0
10	19800	0	0.5	1	0
10	20400	0	0.625	1	0
10	21000	0	1.125	1	0
10	21600	0	0.375	1	0
10	22200	0	0.625	1	0
10	22800	0	0.5	1	0
10	23400	0	0.75	1	0
10	24000	0	0.625	1	0
10	24600	0	0.5	1	0
10	25200	0	0.375	1	0
10	25800	0	0.75	1	0
10	26400	0	0.5	1	0
10	27000	0	0.75	1	0
10	27600	0	0.75	1	0
10	28200	0	0.625	0	0

10	28800	0	0.75	0	0
10	29400	0	1	0	0
10	30000	0	0.25	1	0
10	30600	0	0.625	1	0
10	31200	0	0.625	1	0
10	31800	0	0.625	1	0
10	32400	0	1	1	0
10	33000	0	1	0	0
10	33600	0	1.375	0	1
10	34200	0	1	0	0
10	34800	0	1.625	0	0
10	35400	0	1.125	0	0
10	36000	0	1.25	0	0
10	36600	0	1.75	0	1
10	37200	0	1.5	0	0
10	37800	0	1	0	0
10	38400	0	1.125	0	0
10	39000	0	1.375	0	0
10	39600	0	1	0	0
10	40200	0	1.25	0	0
10	40800	0	1	0	0
10	41400	0	0.875	1	0
10	42000	0	0.875	0	0
10	42600	0	1	0	0
10	43200	0	1.5	0	0
10	43800	0	0.875	0	0
10	44400	0	1	0	0
10	45000	0	1.625	1	1
10	45600	0	2	0	0
10	46200	0	1.125	0	1
10	46800	0	0.5	0	0
10	47400	0	1.125	0	0
10	48000	0	1.25	0	0
10	48600	0	1.125	0	0
10	49200	0	1	0	0
10	49800	0	1.375	0	1
10	50400	0	0.875	0	0
10	51000	0	1.25	0	0
10	51600	0	0.875	0	0
10	52200	0	2.125	0	0
10	52800	0	2.5	0	0
10	53400	0	1.5	0	0
10	54000	0	1.5	0	0
10	54600	0	2.5	0	0
10	55200	0	1.375	0	0
10	55800	0	1.625	0	0
10	56400	0	2.25	0	1
10	57000	0	1.25	0	0

10	57600	0	0.625	1	0
10	58200	0	1	0	0
10	58800	0	2.25	0	0
10	59400	0	2.25	0	1
10	60000	0	1.75	1	1
10	60600	0	1.5	0	0
10	61200	0	1.375	0	0
10	61800	0	2	0	0
10	62400	0	2.125	0	1
10	63000	0	0.75	1	0
10	63600	0	1	0	0
10	64200	0	1.625	0	1
10	64800	0	0.875	0	0
10	65400	0	2.375	0	0
10	66000	0	1.625	0	0
10	66600	0	2.25	0	1
10	67200	0	1.75	0	1
10	67800	0	1	0	0
10	68400	0	1.125	0	0
10	69000	0	2.25	0	0
10	69600	0	2.25	0	0
10	70200	0	1.5	0	0
10	70800	0	2.25	0	0
10	71400	0	1.25	0	0
10	72000	0	1.375	0	0
10	72600	0	1.375	0	0
10	73200	0	2	0	0
10	73800	0	1.375	0	0
10	74400	0	1.375	0	0
10	75000	0	1.625	0	0
10	75600	0	0.875	1	0
10	76200	0	1.125	0	0
10	76800	0	1.25	0	0
10	77400	0	0.875	0	0
10	78000	0	1.25	0	0
10	78600	0	1.5	0	0
10	79200	0	1.125	0	0
10	79800	0	2.25	0	1
10	80400	0	1.5	0	0
10	81000	0	1.375	0	0
10	81600	0	0.625	1	0
10	82200	0	1.875	0	1
10	82800	0	0.875	0	0
10	83400	0	1.75	0	0
10	84000	0	1.875	0	1
10	84600	0	2.625	0	0
10	85200	0	1.75	0	0
10	85800	0	2.125	0	0

10	86400	0	2.5	0	1
11	600	0	.	1	0
11	1200	0	1	1	0
11	1800	0	0.429	1	0
11	2400	0	0.571	0	0
11	3000	0	0.143	1	0
11	3600	0	2.714	1	1
11	4200	0	2.143	1	1
11	4800	0	0.143	1	0
11	5400	0	1.143	1	0
11	6000	0	0.429	1	0
11	6600	0	0.714	0	0
11	7200	0	0.571	1	0
11	7800	0	0.429	1	0
11	8400	0	0.429	1	0
11	9000	0	2	1	1
11	9600	0	2	0	0
11	10200	0	1.286	0	0
11	10800	0	1	1	1
11	11400	0	1	1	1
11	12000	0	2	1	1
11	12600	0	1.143	0	1
11	13200	0	0.857	1	0
11	13800	0	0.857	1	0
11	14400	0	0.429	1	0
11	15000	0	0.571	1	0
11	15600	0	0.429	1	0
11	16200	0	0	1	0
11	16800	0	0.571	0	0
11	17400	0	1.143	1	0
11	18000	0	0.857	1	0
11	18600	0	0.857	1	0
11	19200	0	1.286	0	0
11	19800	0	0.714	1	0
11	20400	0	0.857	1	0
11	21000	0	1.286	1	1
11	21600	0	0.143	1	0
11	22200	0	1.143	1	0
11	22800	0	0.571	1	0
11	23400	0	0.571	1	0
11	24000	0	1.286	0	1
11	24600	0	2	0	0
11	25200	0	1.429	0	1
11	25800	0	1.143	0	0
11	26400	0	1.286	0	1
11	27000	0	1.286	0	0
11	27600	0	1.429	0	1
11	28200	0	1.286	0	1

11	28800	0	2	1	1
11	29400	0	1	1	0
11	30000	0	0.571	1	0
11	30600	0	0.857	0	0
11	31200	0	1	0	0
11	31800	0	0.429	1	0
11	32400	0	0.429	1	0
11	33000	0	2	0	0
11	33600	0	2.714	0	1
11	34200	0	1.143	0	0
11	34800	0	1	1	0
11	35400	0	1.857	0	0
11	36000	0	0.857	0	0
11	36600	0	1.714	0	0
11	37200	0	1.143	0	0
11	37800	0	1.857	0	0
11	38400	0	1.429	0	0
11	39000	0	0.857	0	0
11	39600	0	1.286	0	0
11	40200	0	0.857	0	0
11	40800	0	1.429	0	0
11	41400	0	1.429	0	0
11	42000	0	2	0	1
11	42600	0	1.286	0	0
11	43200	0	1.286	0	0
11	43800	0	0.714	1	0
11	44400	0	1.143	0	0
11	45000	0	1.286	0	0
11	45600	0	1.143	0	0
11	46200	0	0.857	0	0
11	46800	0	2.429	1	1
11	47400	0	2.143	0	0
11	48000	0	2	0	0
11	48600	0	0.571	1	0
11	49200	0	1.143	0	1
11	49800	0	1.143	0	0
11	50400	0	1.286	0	0
11	51000	0	1	0	0
11	51600	0	1.143	0	0
11	52200	0	0.857	0	0
11	52800	0	0.429	1	0
11	53400	0	0.714	1	0
11	54000	0	0.429	1	0
11	54600	0	1	1	0
11	55200	0	0.571	1	0
11	55800	0	0.571	0	0
11	56400	0	0.714	1	0
11	57000	0	1	0	0

11	57600	0	1.143	0	0
11	58200	0	1.286	0	0
11	58800	0	1.286	0	0
11	59400	0	1.286	0	0
11	60000	0	1	0	0
11	60600	0	1.286	0	0
11	61200	0	1.571	1	1
11	61800	0	2.571	1	1
11	62400	0	1.857	0	1
11	63000	0	2.571	0	0
11	63600	0	0.857	0	0
11	64200	0	0.714	0	0
11	64800	0	0.857	0	0
11	65400	0	0.571	1	0
11	66000	0	0.571	0	0
11	66600	0	0.429	1	0
11	67200	0	1.143	1	0
11	67800	0	0.857	1	0
11	68400	0	0.857	1	0
11	69000	0	2	1	1
11	69600	0	1.714	1	1
11	70200	0	1.714	0	0
11	70800	0	1.714	0	0
11	71400	0	3.143	1	1
11	72000	0	0.857	1	0
11	72600	0	0.714	1	0
11	73200	0	0.429	1	0
11	73800	0	1.857	0	0
11	74400	0	1.714	0	0
11	75000	0	0.714	0	0
11	75600	0	1.286	0	0
11	76200	0	1.857	0	0
11	76800	0	1	0	0
11	77400	0	1.429	0	0
11	78000	0	1.286	0	1
11	78600	0	2	0	1
11	79200	0	2.143	0	1
11	79800	0	1.286	0	0
11	80400	0	1.857	0	0
11	81000	0	2.143	0	0
11	81600	0	2	0	0
11	82200	0	1.571	0	0
11	82800	0	2.143	0	1
11	83400	0	1.429	0	0
11	84000	0	2	1	1
11	84600	0	1.571	0	0
11	85200	0	1.143	1	0
11	85800	0	0.857	0	0

11	86400	0	0.571	0	0
12	600	0	0.5	1	0
12	1200	0	0.5	1	0
12	1800	0	0.375	1	0
12	2400	0	0.375	1	0
12	3000	0	0.5	1	0
12	3600	0	0.5	1	0
12	4200	0	0.5	1	0
12	4800	0	0.25	1	0
12	5400	0	0.25	1	0
12	6000	0	0.25	1	0
12	6600	0	0.25	1	0
12	7200	0	0.25	1	0
12	7800	0	0.25	1	0
12	8400	0	0.25	1	0
12	9000	0	0.25	1	0
12	9600	0	0.25	1	0
12	10200	0	0.25	1	0
12	10800	0	0.25	1	0
12	11400	0	0.25	1	0
12	12000	0	0.375	1	0
12	12600	0	0.25	1	0
12	13200	0	1.875	1	1
12	13800	0	1.75	1	1
12	14400	0	1.25	1	1
12	15000	0	1.25	1	1
12	15600	0	1.375	1	1
12	16200	0	1.375	1	1
12	16800	0	0.625	1	0
12	17400	0	0.375	1	0
12	18000	0	0.5	1	0
12	18600	0	0.625	1	0
12	19200	0	1.375	1	1
12	19800	0	1.125	0	0
12	20400	0	1	0	0
12	21000	0	0.5	0	0
12	21600	0	0.5	0	0
12	22200	0	0.625	0	0
12	22800	0	0.5	1	0
12	23400	0	0.25	1	0
12	24000	0	0.875	1	0
12	24600	0	1	1	0
12	25200	0	0.25	1	0
12	25800	0	0.125	1	0
12	26400	0	0.25	1	0
12	27000	0	1.125	1	0
12	27600	0	2.5	1	1
12	28200	0	1	0	0

12	28800	0	0.75	0	0
12	29400	0	0.375	1	0
12	30000	0	1.25	1	0
12	30600	0	2.375	0	1
12	31200	0	2.25	0	0
12	31800	0	0.75	0	0
12	32400	0	1	0	0
12	33000	0	0.625	0	0
12	33600	0	1.25	0	0
12	34200	0	1.375	0	0
12	34800	0	2	0	1
12	35400	0	1.25	0	0
12	36000	0	1.25	0	0
12	36600	0	1.25	0	1
12	37200	0	1.375	0	1
12	37800	0	0.875	0	0
12	38400	0	2	0	0
12	39000	0	1.375	0	0
12	39600	0	1.5	0	0
12	40200	0	1.125	0	0
12	40800	0	1	0	0
12	41400	0	1	0	0
12	42000	0	1.875	0	0
12	42600	0	1.625	0	0
12	43200	0	1	0	0
12	43800	0	2.125	0	1
12	44400	0	1.875	0	0
12	45000	0	1.625	0	0
12	45600	0	1.75	0	0
12	46200	0	2.25	0	0
12	46800	0	2.25	0	0
12	47400	0	1	0	0
12	48000	0	2.125	1	1
12	48600	0	2	0	0
12	49200	0	1.125	0	0
12	49800	0	2	1	1
12	50400	0	0.875	0	0
12	51000	0	1.625	0	0
12	51600	0	1.25	0	0
12	52200	0	1.125	0	0
12	52800	0	1.25	0	0
12	53400	0	1.75	0	0
12	54000	0	1.625	0	0
12	54600	0	1.125	0	1
12	55200	0	3	0	1
12	55800	0	0.75	0	0
12	56400	0	2	1	1
12	57000	0	1.875	0	0

12	57600	0	1.375	0	0
12	58200	0	0.75	1	0
12	58800	0	1.5	1	1
12	59400	0	1.375	0	0
12	60000	0	0.875	0	0
12	60600	0	1.125	0	0
12	61200	0	1.75	0	1
12	61800	0	0.875	0	0
12	62400	0	2	1	1
12	63000	0	1.25	0	0
12	63600	0	1.375	0	0
12	64200	0	1.375	0	0
12	64800	0	0.625	0	0
12	65400	0	1.75	0	0
12	66000	0	0.625	1	0
12	66600	0	1	0	0
12	67200	0	1.25	1	0
12	67800	0	1.5	0	1
12	68400	0	0.75	0	0
12	69000	0	0.875	1	0
12	69600	0	0.625	0	0
12	70200	0	1.25	0	0
12	70800	0	1.625	1	1
12	71400	0	1	0	0
12	72000	0	0.875	1	0
12	72600	0	1.25	0	0
12	73200	0	1.75	0	0
12	73800	0	0.875	0	0
12	74400	0	0.875	1	0
12	75000	0	1.625	0	0
12	75600	0	0.625	0	0
12	76200	0	1.5	0	0
12	76800	0	1.5	0	0
12	77400	0	0.75	0	0
12	78000	0	0.625	0	0
12	78600	0	0.625	1	0
12	79200	0	1.25	1	0
12	79800	0	1.875	1	1
12	80400	0	1.375	0	0
12	81000	0	0.75	1	0
12	81600	0	1.625	1	1
12	82200	0	0.75	1	0
12	82800	0	0.75	1	0
12	83400	0	0.375	1	0
12	84000	0	0.75	1	0
12	84600	0	1.125	1	0
12	85200	0	1.375	1	0
12	85800	0	1.125	1	0

12	86400	0	1.125	1	0
13	600	0	.	1	0
13	1200	0	.	1	0
13	1800	0	0.143	1	0
13	2400	0	0.286	1	0
13	3000	0	2.143	1	1
13	3600	0	1.286	1	1
13	4200	0	1.143	0	0
13	4800	0	1.143	1	0
13	5400	0	1	1	0
13	6000	0	0.286	1	0
13	6600	0	0.857	1	0
13	7200	0	0.143	1	0
13	7800	0	1.143	1	1
13	8400	0	0.714	1	0
13	9000	0	0.714	1	0
13	9600	0	0.714	1	0
13	10200	0	0.857	0	0
13	10800	0	0.857	1	0
13	11400	0	0.286	1	0
13	12000	0	0.429	1	0
13	12600	0	0.429	1	0
13	13200	0	0.571	1	0
13	13800	0	0.286	1	0
13	14400	0	0.143	1	0
13	15000	0	0.429	1	0
13	15600	0	0.286	1	0
13	16200	0	0.286	1	0
13	16800	0	0.571	1	0
13	17400	0	0.429	1	0
13	18000	0	0.429	1	0
13	18600	0	0.429	1	0
13	19200	0	0.286	1	0
13	19800	0	0.571	1	0
13	20400	0	0.286	1	0
13	21000	0	0.286	1	0
13	21600	0	0.429	1	0
13	22200	0	0.429	1	0
13	22800	0	0.286	1	0
13	23400	0	0.286	1	0
13	24000	0	0.286	1	0
13	24600	0	0.714	1	0
13	25200	0	0.429	1	0
13	25800	0	0.429	1	0
13	26400	0	0.286	1	0
13	27000	0	0.857	1	0
13	27600	0	1.286	0	0
13	28200	0	0.571	1	0

13	28800	0	0.714	1	0
13	29400	0	0.429	1	0
13	30000	0	0.714	0	0
13	30600	0	1.143	0	0
13	31200	0	0.571	1	0
13	31800	0	0.714	1	0
13	32400	0	0.857	1	0
13	33000	0	1	1	0
13	33600	0	0.429	1	0
13	34200	0	0.571	1	0
13	34800	0	0.857	1	0
13	35400	0	0.286	1	0
13	36000	0	1.714	1	1
13	36600	0	1.857	1	1
13	37200	0	0.286	1	0
13	37800	0	0.714	1	0
13	38400	0	0.429	1	0
13	39000	0	0.571	0	0
13	39600	0	1.143	0	0
13	40200	0	0.714	1	0
13	40800	0	0.714	1	0
13	41400	0	0.429	1	0
13	42000	0	0.571	1	0
13	42600	0	1	0	0
13	43200	0	1.143	0	1
13	43800	0	0.857	1	0
13	44400	0	1.286	0	0
13	45000	0	0.714	0	0
13	45600	0	1.286	0	1
13	46200	0	1.429	1	0
13	46800	0	0.571	1	0
13	47400	0	1.143	0	0
13	48000	0	0.857	1	0
13	48600	0	1	0	0
13	49200	0	1.143	0	0
13	49800	0	0.857	0	0
13	50400	0	0.571	0	0
13	51000	0	0.571	1	0
13	51600	0	2.429	0	1
13	52200	0	1.143	0	0
13	52800	0	0.571	1	0
13	53400	0	0.571	0	0
13	54000	0	0.286	1	0
13	54600	0	1.286	0	1
13	55200	0	1.571	0	0
13	55800	0	0.571	1	0
13	56400	0	2	0	0
13	57000	0	1.714	1	1

13	57600	0	0.857	0	0
13	58200	0	0.714	0	0
13	58800	0	0.857	0	0
13	59400	0	1.286	0	0
13	60000	0	1.143	0	0
13	60600	0	0.857	0	0
13	61200	0	0.857	1	0
13	61800	0	1.143	0	0
13	62400	0	1.571	0	0
13	63000	0	1.429	0	0
13	63600	0	1.429	0	0
13	64200	0	1.143	0	0
13	64800	0	0.857	0	0
13	65400	0	0.571	1	0
13	66000	0	1.286	0	1
13	66600	0	1	0	0
13	67200	0	1.286	0	0
13	67800	0	0.571	1	0
13	68400	0	1	0	0
13	69000	0	1	0	0
13	69600	0	1	0	0
13	70200	0	1.143	0	1
13	70800	0	1.571	1	1
13	71400	0	1.143	0	0
13	72000	0	0.857	0	0
13	72600	0	1.143	0	0
13	73200	0	2.143	0	1
13	73800	0	1.143	1	0
13	74400	0	1.143	0	0
13	75000	0	0.714	1	0
13	75600	0	1	1	1
13	76200	0	1	1	0
13	76800	0	1	0	0
13	77400	0	0.429	1	0
13	78000	0	1	0	0
13	78600	0	1.714	1	1
13	79200	0	0.714	0	0
13	79800	0	1.143	0	0
13	80400	0	0.571	1	0
13	81000	0	0.429	1	0
13	81600	0	0.286	1	0
13	82200	0	0.286	1	0
13	82800	0	1.143	1	0
13	83400	0	1.286	1	0
13	84000	0	1.143	1	0
13	84600	0	0.429	1	0
13	85200	0	0.857	1	0
13	85800	0	0.857	0	0

13	86400	0	0.571	1	0
14	600	0	0.429	1	0
14	1200	0	0.571	1	0
14	1800	0	1.429	1	1
14	2400	0	0.286	1	0
14	3000	0	0.714	1	0
14	3600	0	1	1	0
14	4200	0	1	1	0
14	4800	0	0.143	1	0
14	5400	0	1	1	0
14	6000	0	0.286	1	0
14	6600	0	1.714	1	1
14	7200	0	1.286	1	1
14	7800	0	0	1	0
14	8400	0	0.857	1	0
14	9000	0	0.857	1	0
14	9600	0	1.714	1	0
14	10200	0	1.429	1	1
14	10800	0	2.286	1	1
14	11400	0	0.429	0	0
14	12000	0	1	1	1
14	12600	0	1	1	1
14	13200	0	1	1	1
14	13800	0	2.143	0	1
14	14400	0	0.571	1	0
14	15000	0	0.714	1	0
14	15600	0	1.429	1	1
14	16200	0	0.143	1	0
14	16800	0	0.429	0	0
14	17400	0	1.429	0	0
14	18000	0	0.857	1	0
14	18600	0	0.714	0	0
14	19200	0	0.571	1	0
14	19800	0	0.571	1	0
14	20400	0	1.429	0	0
14	21000	0	2.571	0	1
14	21600	0	0.571	1	0
14	22200	0	0.571	0	0
14	22800	0	0.714	0	0
14	23400	0	0.429	0	0
14	24000	0	2.143	1	1
14	24600	0	1.571	0	0
14	25200	0	1.286	0	0
14	25800	0	2	0	0
14	26400	0	1	0	0
14	27000	0	1.286	0	0
14	27600	0	1.286	0	1
14	28200	0	2	0	0

14	28800	0	1.857	0	1
14	29400	0	1.857	0	0
14	30000	0	1.143	0	0
14	30600	0	1	0	0
14	31200	0	1.143	0	0
14	31800	0	0.857	1	0
14	32400	0	2.143	0	1
14	33000	0	1.143	0	0
14	33600	0	1.286	0	0
14	34200	0	1.714	0	0
14	34800	0	1.429	0	0
14	35400	0	1.286	0	0
14	36000	0	0.429	0	0
14	36600	0	1.429	0	1
14	37200	0	1.143	0	0
14	37800	0	1.286	0	0
14	38400	0	1.143	0	0
14	39000	0	1.857	0	0
14	39600	0	2	0	0
14	40200	0	1.714	0	0
14	40800	0	1	0	0
14	41400	0	1.429	0	1
14	42000	0	1.429	0	0
14	42600	0	1.429	0	1
14	43200	0	2	0	0
14	43800	0	1	0	0
14	44400	0	1.571	0	0
14	45000	0	0.857	0	0
14	45600	0	1.429	0	1
14	46200	0	1	0	0
14	46800	0	2.143	0	0
14	47400	0	2.143	0	0
14	48000	0	1.143	0	0
14	48600	0	1	1	0
14	49200	0	1.286	0	0
14	49800	0	1	0	0
14	50400	0	1.429	0	0
14	51000	0	2.286	0	0
14	51600	0	1.857	0	0
14	52200	0	3	1	1
14	52800	0	0.571	1	0
14	53400	0	1	0	0
14	54000	0	1	1	0
14	54600	0	1.286	0	0
14	55200	0	0.857	0	0
14	55800	0	1.857	0	0
14	56400	0	0.714	0	0
14	57000	0	1.857	1	1

14	57600	0	1.429	0	1
14	58200	0	1.286	0	0
14	58800	0	1.286	0	0
14	59400	0	1.429	0	1
14	60000	0	0.714	0	0
14	60600	0	1.286	0	0
14	61200	0	0.714	0	0
14	61800	0	0.857	0	0
14	62400	0	1.714	0	1
14	63000	0	2	0	1
14	63600	0	2	1	1
14	64200	0	2.143	0	1
14	64800	0	1	0	0
14	65400	0	1.286	0	0
14	66000	0	1.286	0	1
14	66600	0	3.143	1	1
14	67200	0	0.857	0	0
14	67800	0	0.857	0	0
14	68400	0	1.286	0	0
14	69000	0	0.857	0	0
14	69600	0	1	0	0
14	70200	0	1.286	0	0
14	70800	0	2.143	1	1
14	71400	0	2.143	0	0
14	72000	0	1.571	0	0
14	72600	0	0.714	0	0
14	73200	0	0.857	0	0
14	73800	0	2.143	0	0
14	74400	0	1.429	0	0
14	75000	0	1.429	0	0
14	75600	0	0.857	1	0
14	76200	0	1.143	0	1
14	76800	0	1	0	0
14	77400	0	1.143	0	0
14	78000	0	0.857	0	0
14	78600	0	2.143	0	1
14	79200	0	2.429	0	1
14	79800	0	0.571	1	0
14	80400	0	2.714	1	1
14	81000	0	2	1	1
14	81600	0	0.286	1	0
14	82200	0	1	1	0
14	82800	0	1.286	1	1
14	83400	0	0.571	1	0
14	84000	0	0.429	1	0
14	84600	0	0.429	1	0
14	85200	0	0.429	1	0
14	85800	0	0.429	1	0

14	86400	0	0.429	1	0
15	600	0	.	1	0
15	1200	0	.	1	0
15	1800	0	.	1	0
15	2400	0	.	1	0
15	3000	0	.	1	0
15	3600	0	.	1	0
15	4200	0	.	1	0
15	4800	0	1.125	1	1
15	5400	0	1.125	1	1
15	6000	0	2.125	1	1
15	6600	0	0.125	1	0
15	7200	0	0.125	1	0
15	7800	0	0.125	1	0
15	8400	0	0.125	1	0
15	9000	0	0.125	1	0
15	9600	0	0.375	0	0
15	10200	0	1	1	0
15	10800	0	1.25	1	0
15	11400	0	0.75	1	0
15	12000	0	1	1	0
15	12600	0	1	1	0
15	13200	0	0.875	0	0
15	13800	0	1.75	1	1
15	14400	0	1	1	0
15	15000	0	0.875	1	0
15	15600	0	0.875	1	0
15	16200	0	0.375	1	0
15	16800	0	0.25	1	0
15	17400	0	0.5	0	0
15	18000	0	0.5	0	0
15	18600	0	1.125	0	0
15	19200	0	1.125	0	0
15	19800	0	0.625	0	0
15	20400	0	1	0	0
15	21000	0	0.25	1	0
15	21600	0	0.625	0	0
15	22200	0	2.25	1	1
15	22800	0	0.875	1	0
15	23400	0	1	1	0
15	24000	0	1	1	0
15	24600	0	0.875	0	0
15	25200	0	0.5	0	0
15	25800	0	0.625	0	0
15	26400	0	0.625	0	0
15	27000	0	1.375	0	0
15	27600	0	1.25	0	0
15	28200	0	1.25	0	0

15	28800	0	1.25	0	0
15	29400	0	1.375	0	0
15	30000	0	2.125	0	0
15	30600	0	1.625	0	0
15	31200	0	1.5	0	0
15	31800	0	1.375	0	0
15	32400	0	0.75	0	0
15	33000	0	0.875	0	0
15	33600	0	0.75	0	0
15	34200	0	1.625	0	0
15	34800	0	1.375	0	0
15	35400	0	0.375	1	0
15	36000	0	1.125	0	0
15	36600	0	1.375	0	0
15	37200	0	2.875	0	0
15	37800	0	1	0	0
15	38400	0	0.75	0	0
15	39000	0	2	0	1
15	39600	0	1.625	0	0
15	40200	0	1.25	0	0
15	40800	0	0.875	0	0
15	41400	0	1	0	0
15	42000	0	1.125	0	0
15	42600	0	1.625	0	1
15	43200	0	1.75	0	0
15	43800	0	1.25	0	0
15	44400	0	1	0	0
15	45000	0	1	0	0
15	45600	0	1.5	0	0
15	46200	0	1.25	0	0
15	46800	0	1.5	0	0
15	47400	0	0.75	0	0
15	48000	0	1	0	0
15	48600	0	1.5	0	0
15	49200	0	1.625	0	0
15	49800	0	1.125	1	0
15	50400	0	1.25	1	0
15	51000	0	0.75	1	0
15	51600	0	0.875	0	0
15	52200	0	0.875	1	0
15	52800	0	1	1	0
15	53400	0	1.125	1	0
15	54000	0	1.125	1	0
15	54600	0	0.75	1	0
15	55200	0	1	0	0
15	55800	0	1.625	0	0
15	56400	0	0.875	0	0
15	57000	0	1.375	0	0

15	57600	0	1.625	0	0
15	58200	0	0.875	0	0
15	58800	0	1.75	0	1
15	59400	0	1	0	0
15	60000	0	0.625	1	0
15	60600	0	1	0	0
15	61200	0	1.375	0	0
15	61800	0	1.75	0	0
15	62400	0	2	0	0
15	63000	0	0.875	0	0
15	63600	0	1.25	0	0
15	64200	0	0.875	0	0
15	64800	0	1.25	0	0
15	65400	0	1.375	0	0
15	66000	0	0.875	0	0
15	66600	0	1.25	0	0
15	67200	0	0.75	0	0
15	67800	0	0.75	0	0
15	68400	0	1.5	0	0
15	69000	0	1.25	0	0
15	69600	0	1.125	0	0
15	70200	0	0.75	0	0
15	70800	0	0.75	0	0
15	71400	0	0.5	1	0
15	72000	0	1.125	0	0
15	72600	0	1.5	0	0
15	73200	0	1.25	0	0
15	73800	0	1	0	0
15	74400	0	1.375	0	0
15	75000	0	0.875	0	0
15	75600	0	1.125	1	0
15	76200	0	1	0	0
15	76800	0	0.875	0	0
15	77400	0	1.375	0	0
15	78000	0	1.375	0	1
15	78600	0	2.5	1	1
15	79200	0	1.375	0	0
15	79800	0	1	0	0
15	80400	0	1.25	0	0
15	81000	0	1.125	0	0
15	81600	0	1.5	0	0
15	82200	0	2.25	0	0
15	82800	0	1.5	0	0
15	83400	0	1.25	0	0
15	84000	0	1	1	0
15	84600	0	1.25	1	0
15	85200	0	0.5	1	0
15	85800	0	1.125	0	0

15	86400	0	2.625	0	1
16	600	1	.	1	0
16	1200	1	1.167	0	1
16	1800	1	0.5	0	0
16	2400	1	0.375	1	0
16	3000	1	0.875	1	0
16	3600	1	0.875	1	0
16	4200	1	0.375	1	0
16	4800	1	1	0	0
16	5400	1	1.75	1	1
16	6000	1	1.5	0	0
16	6600	1	0.875	1	0
16	7200	1	0.875	1	0
16	7800	1	1	0	0
16	8400	1	1	0	0
16	9000	1	1.5	0	0
16	9600	1	0.625	0	0
16	10200	1	0.625	0	0
16	10800	1	0.625	0	0
16	11400	1	0.5	1	0
16	12000	1	0.625	1	0
16	12600	1	0.625	0	0
16	13200	1	0.625	1	0
16	13800	1	0.625	1	0
16	14400	1	1.75	1	1
16	15000	1	1.875	0	1
16	15600	1	1.5	1	1
16	16200	1	1.5	1	1
16	16800	1	1.625	1	1
16	17400	1	1.625	1	1
16	18000	1	1.75	1	1
16	18600	1	1.75	1	1
16	19200	1	1.75	1	1
16	19800	1	1.75	1	1
16	20400	1	1.875	1	1
16	21000	1	1.625	0	0
16	21600	1	2.125	0	1
16	22200	1	2.125	0	1
16	22800	1	2	1	1
16	23400	1	2	1	1
16	24000	1	2	1	1
16	24600	1	2	1	1
16	25200	1	2.125	1	1
16	25800	1	1.125	0	0
16	26400	1	0.5	0	0
16	27000	1	0.75	1	0
16	27600	1	1.625	0	0
16	28200	1	1.375	0	0

16	28800	1	2.375	0	0
16	29400	1	2	0	0
16	30000	1	1.375	0	0
16	30600	1	1.875	0	0
16	31200	1	0.75	1	0
16	31800	1	1	1	0
16	32400	1	1.375	0	0
16	33000	1	0.75	1	0
16	33600	1	1.875	1	1
16	34200	1	1.875	0	0
16	34800	1	2.125	0	1
16	35400	1	0.625	1	0
16	36000	1	1.75	0	0
16	36600	1	0.625	1	0
16	37200	1	1.125	1	0
16	37800	1	2.125	1	1
16	38400	1	2.375	0	1
16	39000	1	0.875	0	0
16	39600	1	2.75	0	1
16	40200	1	2.25	0	0
16	40800	1	1.5	0	0
16	41400	1	1.875	0	0
16	42000	1	1.375	0	0
16	42600	1	1.625	0	0
16	43200	1	2	0	0
16	43800	1	2.125	0	1
16	44400	1	1	0	0
16	45000	1	1.625	1	1
16	45600	1	2.625	0	1
16	46200	1	0.875	0	0
16	46800	1	1.125	0	0
16	47400	1	1.25	0	0
16	48000	1	2.125	0	1
16	48600	1	1.375	0	0
16	49200	1	2.25	0	1
16	49800	1	2	0	0
16	50400	1	0.875	0	0
16	51000	1	1.875	0	0
16	51600	1	1.125	0	0
16	52200	1	0.875	0	0
16	52800	1	0.75	0	0
16	53400	1	1.875	0	0
16	54000	1	1.875	0	0
16	54600	1	2.25	0	0
16	55200	1	1.25	1	1
16	55800	1	1.25	0	0
16	56400	1	2	1	1
16	57000	1	1.625	0	0

16	57600	1	1.5	1	1
16	58200	1	1.125	0	0
16	58800	1	2.75	1	1
16	59400	1	1.625	0	0
16	60000	1	1.625	0	0
16	60600	1	2	1	1
16	61200	1	2.875	0	1
16	61800	1	2.875	1	1
16	62400	1	2.125	1	1
16	63000	1	1.125	0	0
16	63600	1	1.375	0	0
16	64200	1	0.875	0	0
16	64800	1	1.5	0	0
16	65400	1	0.875	0	0
16	66000	1	1.375	0	0
16	66600	1	2.125	0	1
16	67200	1	1.875	0	0
16	67800	1	1	0	0
16	68400	1	1.375	0	0
16	69000	1	0.75	0	0
16	69600	1	1.625	0	0
16	70200	1	1.25	1	1
16	70800	1	2.5	0	1
16	71400	1	1.375	0	1
16	72000	1	0.875	1	0
16	72600	1	1.625	1	1
16	73200	1	1	0	0
16	73800	1	1.125	1	0
16	74400	1	0.875	0	0
16	75000	1	1.75	0	0
16	75600	1	1.5	1	1
16	76200	1	1.75	1	1
16	76800	1	1.5	1	1
16	77400	1	1.25	0	0
16	78000	1	0.875	0	0
16	78600	1	1.625	0	0
16	79200	1	0.625	1	0
16	79800	1	1.125	1	0
16	80400	1	0.625	1	0
16	81000	1	0.5	1	0
16	81600	1	0.5	1	0
16	82200	1	0.75	1	0
16	82800	1	0.75	1	0
16	83400	1	0.75	1	0
16	84000	1	0.75	1	0
16	84600	1	0.75	1	0
16	85200	1	0.75	1	0
16	85800	1	0.875	1	0

16	86400	1	0.875	1	0
17	600	1	1	1	0
17	1200	1	0.25	1	0
17	1800	1	2	1	1
17	2400	1	2.125	1	1
17	3000	1	2.125	1	1
17	3600	1	2.125	1	1
17	4200	1	2.125	1	1
17	4800	1	2.125	1	1
17	5400	1	1.875	1	1
17	6000	1	1.875	1	1
17	6600	1	1.875	1	1
17	7200	1	1.875	1	1
17	7800	1	1.75	1	1
17	8400	1	2.5	1	1
17	9000	1	2.25	0	0
17	9600	1	2.5	1	1
17	10200	1	2.5	1	1
17	10800	1	2.75	1	1
17	11400	1	2.375	0	1
17	12000	1	2.5	1	1
17	12600	1	2.375	1	1
17	13200	1	2.125	1	1
17	13800	1	1.75	0	0
17	14400	1	2	1	1
17	15000	1	2	1	1
17	15600	1	2.125	1	1
17	16200	1	1.25	0	1
17	16800	1	1.25	0	1
17	17400	1	1.25	1	1
17	18000	1	1.125	1	1
17	18600	1	0.875	1	0
17	19200	1	0.875	1	0
17	19800	1	0.875	1	0
17	20400	1	1.25	1	1
17	21000	1	1.375	1	1
17	21600	1	1.375	0	1
17	22200	1	0.25	1	0
17	22800	1	1.25	1	1
17	23400	1	1.25	1	1
17	24000	1	1.125	0	1
17	24600	1	0.75	1	0
17	25200	1	1.375	0	0
17	25800	1	1.5	0	1
17	26400	1	0.75	1	0
17	27000	1	0.875	0	0
17	27600	1	1.25	1	0
17	28200	1	0.875	1	0

17	28800	1	2.375	0	1
17	29400	1	2	1	1
17	30000	1	1.125	0	1
17	30600	1	2.25	0	0
17	31200	1	3.375	1	1
17	31800	1	0.625	1	0
17	32400	1	0.875	1	0
17	33000	1	1.5	0	0
17	33600	1	0.75	1	0
17	34200	1	2.375	0	1
17	34800	1	2.625	1	1
17	35400	1	1.375	0	0
17	36000	1	1.5	0	0
17	36600	1	2.25	0	0
17	37200	1	2	0	0
17	37800	1	2.25	0	1
17	38400	1	0.75	1	0
17	39000	1	1.75	0	0
17	39600	1	1.75	0	0
17	40200	1	2.125	0	0
17	40800	1	1.375	0	0
17	41400	1	1	0	0
17	42000	1	2	0	1
17	42600	1	0.875	1	0
17	43200	1	0.625	1	0
17	43800	1	2	0	0
17	44400	1	2.125	1	1
17	45000	1	1.375	1	1
17	45600	1	2.625	0	1
17	46200	1	2	1	1
17	46800	1	1.75	1	1
17	47400	1	2.25	1	1
17	48000	1	1.375	0	0
17	48600	1	1.75	1	1
17	49200	1	0.875	0	0
17	49800	1	0.75	1	0
17	50400	1	1.125	0	0
17	51000	1	1	0	0
17	51600	1	2.5	0	1
17	52200	1	1.375	0	0
17	52800	1	1.25	0	0
17	53400	1	1.25	0	0
17	54000	1	2.25	0	0
17	54600	1	1.625	0	0
17	55200	1	0.75	1	0
17	55800	1	1.875	0	1
17	56400	1	1.125	1	0
17	57000	1	1.75	0	0

17	57600	1	1.875	0	0
17	58200	1	1.25	0	0
17	58800	1	0.75	0	0
17	59400	1	0.75	1	0
17	60000	1	1.25	0	1
17	60600	1	2	0	1
17	61200	1	1.5	0	1
17	61800	1	2.25	0	1
17	62400	1	0.25	1	0
17	63000	1	1.375	0	1
17	63600	1	0.625	0	0
17	64200	1	0.875	1	0
17	64800	1	0.5	1	0
17	65400	1	0.625	1	0
17	66000	1	0.875	0	0
17	66600	1	2.125	1	1
17	67200	1	1.125	0	0
17	67800	1	1.875	0	0
17	68400	1	0.75	1	0
17	69000	1	0.75	0	0
17	69600	1	1.25	0	0
17	70200	1	1.375	0	0
17	70800	1	1	1	0
17	71400	1	0.625	1	0
17	72000	1	1.875	1	1
17	72600	1	1	0	0
17	73200	1	1.75	1	1
17	73800	1	1.625	1	1
17	74400	1	1.75	0	0
17	75000	1	1.25	0	0
17	75600	1	0.75	1	0
17	76200	1	1.75	1	1
17	76800	1	0.75	1	0
17	77400	1	0.5	1	0
17	78000	1	0.5	1	0
17	78600	1	1.625	1	1
17	79200	1	0.625	1	0
17	79800	1	0.375	1	0
17	80400	1	1.125	1	0
17	81000	1	0.375	1	0
17	81600	1	0.75	1	0
17	82200	1	0.5	1	0
17	82800	1	1.125	1	0
17	83400	1	1.125	1	0
17	84000	1	0.75	1	0
17	84600	1	0.875	1	0
17	85200	1	0.875	1	0
17	85800	1	0.875	1	0

17	86400	1	0.875	1	0
18	600	1	0.333	1	0
18	1200	1	0.429	1	0
18	1800	1	0.286	1	0
18	2400	1	0.286	1	0
18	3000	1	0.143	1	0
18	3600	1	0.143	1	0
18	4200	1	0.286	1	0
18	4800	1	1.857	1	1
18	5400	1	0.429	0	0
18	6000	1	0.429	0	0
18	6600	1	0.25	1	0
18	7200	1	0.25	1	0
18	7800	1	0.375	0	0
18	8400	1	0.375	1	0
18	9000	1	0.75	0	0
18	9600	1	0.5	0	0
18	10200	1	0.5	0	0
18	10800	1	0.5	0	0
18	11400	1	0.5	0	0
18	12000	1	0.5	0	0
18	12600	1	0.5	0	0
18	13200	1	0.375	1	0
18	13800	1	0.375	0	0
18	14400	1	0.5	0	0
18	15000	1	0.375	1	0
18	15600	1	0.375	0	0
18	16200	1	0.375	1	0
18	16800	1	0.625	1	0
18	17400	1	0.375	1	0
18	18000	1	0.75	0	0
18	18600	1	0.375	1	0
18	19200	1	1.875	1	1
18	19800	1	0.625	0	0
18	20400	1	1	1	0
18	21000	1	1.125	1	1
18	21600	1	1.125	1	1
18	22200	1	1.125	1	1
18	22800	1	1.125	1	0
18	23400	1	1.125	1	0
18	24000	1	1.125	1	0
18	24600	1	1	1	0
18	25200	1	2.125	1	1
18	25800	1	1.125	0	0
18	26400	1	1.5	0	0
18	27000	1	2	1	1
18	27600	1	2	0	0
18	28200	1	0.5	1	0

18	28800	1	1.125	1	0
18	29400	1	0.375	1	0
18	30000	1	1.875	0	0
18	30600	1	0.875	0	0
18	31200	1	1.875	0	1
18	31800	1	1.125	1	0
18	32400	1	1.625	0	1
18	33000	1	1	0	0
18	33600	1	1.75	0	0
18	34200	1	1.875	0	1
18	34800	1	1.25	1	0
18	35400	1	0.5	1	0
18	36000	1	1.375	0	0
18	36600	1	0.75	1	0
18	37200	1	0.625	1	0
18	37800	1	1.25	0	0
18	38400	1	2.25	0	1
18	39000	1	2.5	1	1
18	39600	1	3	1	1
18	40200	1	3	1	1
18	40800	1	2.875	0	1
18	41400	1	0.125	1	0
18	42000	1	1.75	1	1
18	42600	1	1.625	1	1
18	43200	1	1.5	0	0
18	43800	1	2.375	0	1
18	44400	1	2.625	1	1
18	45000	1	1	0	0
18	45600	1	1.625	0	0
18	46200	1	1.5	1	1
18	46800	1	0.625	1	0
18	47400	1	1.25	1	0
18	48000	1	2.25	0	1
18	48600	1	1.25	0	0
18	49200	1	2.125	0	0
18	49800	1	1	0	0
18	50400	1	1.375	1	0
18	51000	1	1.5	1	1
18	51600	1	1.125	0	0
18	52200	1	1.25	1	0
18	52800	1	0.75	0	0
18	53400	1	0.625	0	0
18	54000	1	0.5	1	0
18	54600	1	1.625	1	1
18	55200	1	1.625	0	0
18	55800	1	1.625	0	0
18	56400	1	1.25	0	1
18	57000	1	1.125	0	0

18	57600	1	0.625	0	0
18	58200	1	1.125	0	0
18	58800	1	0.875	0	0
18	59400	1	1.75	1	1
18	60000	1	1.125	0	0
18	60600	1	1	0	0
18	61200	1	1.125	0	0
18	61800	1	0.875	0	0
18	62400	1	0.875	0	0
18	63000	1	1.875	1	1
18	63600	1	0.875	1	0
18	64200	1	1.375	0	0
18	64800	1	1	1	0
18	65400	1	1.25	0	0
18	66000	1	1	0	0
18	66600	1	1.875	0	0
18	67200	1	0.875	1	0
18	67800	1	1.875	1	1
18	68400	1	1.625	1	1
18	69000	1	1.625	0	0
18	69600	1	1	0	0
18	70200	1	1.125	0	0
18	70800	1	2.625	0	1
18	71400	1	1.25	1	0
18	72000	1	1	0	0
18	72600	1	1	0	0
18	73200	1	1	0	0
18	73800	1	1.375	0	0
18	74400	1	1	0	0
18	75000	1	0.875	0	0
18	75600	1	0.5	1	0
18	76200	1	2.875	0	0
18	76800	1	1.5	0	0
18	77400	1	1.5	0	1
18	78000	1	1.375	0	0
18	78600	1	0.75	0	0
18	79200	1	1.75	0	0
18	79800	1	0.375	1	0
18	80400	1	0.375	1	0
18	81000	1	0.375	1	0
18	81600	1	0.375	1	0
18	82200	1	0.375	1	0
18	82800	1	0.375	1	0
18	83400	1	0.375	1	0
18	84000	1	0.375	1	0
18	84600	1	0.375	1	0
18	85200	1	0.375	1	0
18	85800	1	0.375	1	0

18	86400	1	0.375	1	0
19	600	1	.	1	0
19	1200	1	2.143	1	1
19	1800	1	1.875	1	1
19	2400	1	1.25	0	1
19	3000	1	2.125	1	1
19	3600	1	2.25	1	1
19	4200	1	1.125	1	1
19	4800	1	0.25	1	0
19	5400	1	0.25	1	0
19	6000	1	0.25	1	0
19	6600	1	1.125	0	1
19	7200	1	0.25	1	0
19	7800	1	0.25	1	0
19	8400	1	2	1	1
19	9000	1	2	1	1
19	9600	1	2	1	1
19	10200	1	2	1	1
19	10800	1	2	1	1
19	11400	1	1.875	1	1
19	12000	1	2.125	1	1
19	12600	1	2	0	0
19	13200	1	2.25	0	1
19	13800	1	1.875	1	1
19	14400	1	1.625	1	0
19	15000	1	2	0	0
19	15600	1	1.25	0	0
19	16200	1	0.625	0	0
19	16800	1	1.375	1	1
19	17400	1	1.375	1	1
19	18000	1	1.375	1	1
19	18600	1	1.875	1	1
19	19200	1	1	1	1
19	19800	1	2	1	1
19	20400	1	0.5	1	0
19	21000	1	2.375	0	1
19	21600	1	2.5	0	1
19	22200	1	2.625	0	1
19	22800	1	2.125	0	1
19	23400	1	2.625	1	1
19	24000	1	2.375	1	1
19	24600	1	2.25	0	1
19	25200	1	2.125	0	0
19	25800	1	1.5	1	1
19	26400	1	1.625	1	1
19	27000	1	1.625	1	1
19	27600	1	1.625	1	1
19	28200	1	1.625	1	1

19	28800	1	2	1	1
19	29400	1	1	0	0
19	30000	1	1	0	0
19	30600	1	2.25	0	0
19	31200	1	1.875	0	0
19	31800	1	2	0	0
19	32400	1	2.125	0	0
19	33000	1	1.5	0	0
19	33600	1	2.375	0	0
19	34200	1	2.75	0	1
19	34800	1	0.875	0	0
19	35400	1	1.125	0	0
19	36000	1	1.375	1	0
19	36600	1	1.75	0	0
19	37200	1	2.125	0	0
19	37800	1	1.375	0	0
19	38400	1	0.875	0	0
19	39000	1	1.375	0	0
19	39600	1	1.875	0	0
19	40200	1	2.5	0	1
19	40800	1	1.125	0	0
19	41400	1	3.125	1	1
19	42000	1	1.25	0	0
19	42600	1	1.25	0	0
19	43200	1	2.5	0	1
19	43800	1	2.25	0	0
19	44400	1	1.375	0	0
19	45000	1	0.75	1	0
19	45600	1	0.75	1	0
19	46200	1	1	0	0
19	46800	1	1.375	0	1
19	47400	1	2	1	1
19	48000	1	1.25	0	0
19	48600	1	0.875	0	0
19	49200	1	1.5	0	0
19	49800	1	1.875	1	1
19	50400	1	2.125	1	1
19	51000	1	1.5	1	1
19	51600	1	1.375	1	1
19	52200	1	2.75	1	1
19	52800	1	1.5	0	0
19	53400	1	1.5	0	1
19	54000	1	1.375	0	0
19	54600	1	2.375	0	1
19	55200	1	1.75	0	0
19	55800	1	1.625	0	0
19	56400	1	2.25	0	0
19	57000	1	2.25	0	0

19	57600	1	1.25	0	0
19	58200	1	1.25	0	0
19	58800	1	1.5	0	0
19	59400	1	1.75	0	0
19	60000	1	1.375	0	0
19	60600	1	1.5	0	0
19	61200	1	2	0	0
19	61800	1	1.25	0	0
19	62400	1	2.25	0	0
19	63000	1	2.25	0	1
19	63600	1	1.625	0	0
19	64200	1	1.25	0	0
19	64800	1	0.875	0	0
19	65400	1	0.875	0	0
19	66000	1	2.5	0	1
19	66600	1	1.875	0	0
19	67200	1	0.875	0	0
19	67800	1	1.75	0	0
19	68400	1	1.5	0	1
19	69000	1	1	0	0
19	69600	1	2.375	0	1
19	70200	1	1.875	1	1
19	70800	1	2.375	0	1
19	71400	1	1.75	0	0
19	72000	1	0.625	1	0
19	72600	1	0.625	1	0
19	73200	1	2.625	0	1
19	73800	1	2.5	0	0
19	74400	1	2.375	0	0
19	75000	1	2.375	0	0
19	75600	1	1.125	0	0
19	76200	1	1	0	0
19	76800	1	2.625	0	1
19	77400	1	2.625	0	0
19	78000	1	2.25	0	0
19	78600	1	1.375	0	0
19	79200	1	1.875	0	0
19	79800	1	1.125	0	0
19	80400	1	1.125	0	0
19	81000	1	1.375	0	0
19	81600	1	1.625	0	0
19	82200	1	1	0	0
19	82800	1	2.75	0	0
19	83400	1	2.25	0	0
19	84000	1	1.5	0	0
19	84600	1	0.875	0	0
19	85200	1	1.25	0	0
19	85800	1	1.875	0	0

19	86400	1	2	0	0
20	600	1	.	1	0
20	1200	1	0.714	1	0
20	1800	1	1.714	1	1
20	2400	1	1.857	1	1
20	3000	1	1.714	1	1
20	3600	1	0.286	1	0
20	4200	1	0.286	1	0
20	4800	1	1.714	1	1
20	5400	1	1.857	1	1
20	6000	1	0.875	1	0
20	6600	1	1.75	1	0
20	7200	1	1.875	1	1
20	7800	1	1.75	0	0
20	8400	1	1.875	0	0
20	9000	1	2	1	1
20	9600	1	1.625	0	0
20	10200	1	1.875	1	1
20	10800	1	1.625	1	1
20	11400	1	1.625	1	0
20	12000	1	1.625	1	1
20	12600	1	2	1	1
20	13200	1	2	1	1
20	13800	1	2.125	1	1
20	14400	1	2.125	1	1
20	15000	1	1.875	0	0
20	15600	1	2.125	0	0
20	16200	1	1.75	0	0
20	16800	1	2	1	1
20	17400	1	1.75	0	0
20	18000	1	1.75	1	1
20	18600	1	2.125	0	1
20	19200	1	0.375	1	0
20	19800	1	0.5	1	0
20	20400	1	0.5	1	0
20	21000	1	0.625	1	0
20	21600	1	0.625	1	0
20	22200	1	0.5	1	0
20	22800	1	0.625	1	0
20	23400	1	0.625	1	0
20	24000	1	0.625	1	0
20	24600	1	0.75	0	0
20	25200	1	0.5	1	0
20	25800	1	0.625	0	0
20	26400	1	1.125	1	0
20	27000	1	1.5	0	0
20	27600	1	1.125	0	0
20	28200	1	1	0	0

20	28800	1	1.125	0	0
20	29400	1	1.125	0	1
20	30000	1	0.875	0	0
20	30600	1	1.5	1	0
20	31200	1	0.75	1	0
20	31800	1	1.125	0	0
20	32400	1	1	0	0
20	33000	1	1.125	0	0
20	33600	1	1.75	0	0
20	34200	1	0.625	0	0
20	34800	1	1.5	0	0
20	35400	1	1.25	0	0
20	36000	1	1.625	0	1
20	36600	1	1.625	0	0
20	37200	1	1.125	0	0
20	37800	1	1.5	0	0
20	38400	1	1.375	0	0
20	39000	1	0.625	1	0
20	39600	1	0.75	1	0
20	40200	1	1.125	0	0
20	40800	1	0.75	0	0
20	41400	1	0.875	0	0
20	42000	1	1.25	1	0
20	42600	1	1.25	0	0
20	43200	1	1.625	1	1
20	43800	1	2.375	0	0
20	44400	1	0.875	0	0
20	45000	1	1.75	1	1
20	45600	1	2.375	0	0
20	46200	1	2.125	0	1
20	46800	1	1.625	1	1
20	47400	1	0.75	0	0
20	48000	1	1.5	0	0
20	48600	1	1.75	0	0
20	49200	1	0.625	1	0
20	49800	1	1.25	0	0
20	50400	1	1	0	0
20	51000	1	0.875	1	0
20	51600	1	0.875	1	0
20	52200	1	2.25	0	1
20	52800	1	1.75	0	0
20	53400	1	2.25	0	1
20	54000	1	1.5	0	0
20	54600	1	2.25	0	1
20	55200	1	1.875	1	1
20	55800	1	1.875	0	0
20	56400	1	1.5	0	0
20	57000	1	1.75	0	0

20	57600	1	1.375	0	0
20	58200	1	1.5	0	1
20	58800	1	1	0	0
20	59400	1	1	0	0
20	60000	1	1.25	0	0
20	60600	1	2	0	0
20	61200	1	2.375	0	1
20	61800	1	1.375	0	0
20	62400	1	1	0	0
20	63000	1	1.5	0	0
20	63600	1	2	0	0
20	64200	1	2.125	0	1
20	64800	1	1.25	1	1
20	65400	1	1.125	1	0
20	66000	1	2	0	0
20	66600	1	2.125	0	1
20	67200	1	2.125	0	1
20	67800	1	1.625	0	0
20	68400	1	2.25	0	1
20	69000	1	2.5	0	0
20	69600	1	1.875	1	1
20	70200	1	1.5	0	0
20	70800	1	2.375	0	1
20	71400	1	2.125	0	0
20	72000	1	1.75	0	0
20	72600	1	1.125	1	0
20	73200	1	1.75	0	0
20	73800	1	1.75	0	0
20	74400	1	1.875	0	0
20	75000	1	2.125	0	1
20	75600	1	1	0	0
20	76200	1	1.25	0	0
20	76800	1	1.25	0	0
20	77400	1	0.875	0	0
20	78000	1	0.75	1	0
20	78600	1	2.125	1	1
20	79200	1	0.75	0	0
20	79800	1	1	0	0
20	80400	1	2	1	1
20	81000	1	1.75	0	0
20	81600	1	0.625	1	0
20	82200	1	0.75	1	0
20	82800	1	0.75	1	0
20	83400	1	0.375	1	0
20	84000	1	1.625	1	1
20	84600	1	0.75	1	0
20	85200	1	0.875	0	0
20	85800	1	0.875	1	0

20	86400	1	0.875	1	0
